# Life history is a key factor explaining functional trait diversity among subtropical grasses, and its influence differs between C_3_ and C_4_ species

**DOI:** 10.1093/jxb/ery462

**Published:** 2019-02-11

**Authors:** Hui Liu, Samuel H Taylor, Qiuyuan Xu, Yixue Lin, Hao Hou, Guilin Wu, Qing Ye

**Affiliations:** 1 Key Laboratory of Vegetation Restoration and Management of Degraded Ecosystems, Guangdong Provincial Key Laboratory of Applied Botany, South China Botanical Garden, Chinese Academy of Sciences, Guangzhou, China; 2 Lancaster Environment Centre, Lancaster University, Lancaster, UK; 3 College of Life Science, University of Chinese Academy of Sciences, Beijing, China

**Keywords:** C_4_ photosynthesis, climatic niche, functional traits, hydraulic conductance, leaf and stem anatomy, phylogeny, Poaceae, seasonality

## Abstract

Life history and photosynthetic type both affect the economics of leaf physiological function. Annual plants have lower tissue densities and resource-use efficiencies than perennials, while C_4_ photosynthesis, facilitated in grasses by specific changes in leaf anatomy, improves photosynthetic efficiency and water-use efficiency, especially in hot climates. This study aimed to determine whether C_4_ photosynthesis affects differences in functional traits between annual and perennial species. We measured 26 traits and characterised niche descriptors for 42 grasses from subtropical China. Differences in the majority of traits were explained by life history. The ranges of annual species (particularly C_4_ annuals) extended to regions with greater temperature seasonality and lower precipitation, and annuals had less-negative turgor-loss points, higher specific leaf areas, and lower water-use efficiencies, stomatal conductances, and leaf areas per stem area than perennials. Photosynthetic type largely affected leaf physiology as expected, but interacted with life history in determining specific traits. Leaf hydraulic conductance was intermediate in perennials, highest in C_4_-annuals, and lowest in C_3_-annuals. Densities of stomata and stem vessels were similar across C_3_-perennials and C_4_ species, but stomatal densities were lower and stem vessel densities higher in C_3_-annuals. Phylogenetic principal component analysis confirmed that in this subtropical environment life history is the predominant axis separating species, and annuals and perennials were more different within C_3_ than C_4_ grasses. The interplay between life history and photosynthetic type may be an overlooked factor in shaping the physiological ecology of grasses.

## Teaser

Life history explains most contrasts in functional traits and climatic niches in subtropical grasses from China, and differentiation between annual and perennial species is greater within C_3_ than C_4_ grasses.

## Introduction

Leaf physiology and the economics of leaf resource use, including water use, are key constraints on plant performance and ecological strategies. Annual and perennial life histories are linked with a ‘fast–slow’ leaf economic spectrum, in which short life spans are linked with fast resource acquisition and less efficient resource use (Wright *et al.*, 2004; Reich, 2014; Díaz *et al.*, 2016). Leaf economics also differ between C_3_ and C_4_ plants. C_4_ photosynthesis increases photosynthetic efficiency and is commonly associated with changes in vascular spacing (Sage, 2004; Christin and Osborne, 2014), which should impact the relative costs of leaf construction (Niinemets *et al.*, 2007). C_4_ photosynthesis has had dramatic effects on the macroevolution and macroecology of plants (Ehleringer *et al.*, 1997; Sage, 2004; Edwards *et al.*, 2010; Griffith *et al.*, 2015), but the functional consequences of C_4_ photosynthesis have commonly been treated as independent of the unique features of species and lineages that utilise them (Edwards *et al.*, 2007). Plant lineages such as the grasses, that show multiple independent origins of C_4_ photosynthesis, provide opportunities to address the impact that photosynthetic type has had on the physiological performance and ecological strategies exploited by plants while accounting for lineage-specific differences (Edwards *et al.*, 2010).

Plant physiologists and ecologists have been fascinated by the physiological contrast between C_3_ and C_4_ plants since the discovery of C_4_ photosynthesis in the mid-20th century (Slack and Hatch, 1967; Osmond *et al.*, 1982; Pearcy and Ehleringer, 1984; Ehleringer and Monson, 1993; Ehleringer *et al.*, 1997; Sage *et al.*, 2012). In circumstances where higher temperatures and/or low CO_2_ availability limit photosynthesis by exacerbating inefficiencies associated with photorespiration, C_4_ photosynthesis improves the rate and efficiency of net CO_2_ assimilation (*A*) compared with C_3_ photosynthesis (Ehleringer and Pearcy, 1983; Sage *et al.*, 2012). The evolution of C_4_ grasses has therefore been linked with physiological advantages under low inter-glacial atmospheric CO_2_ concentrations (Ehleringer *et al.*, 1997; Christin *et al.*, 2008), higher leaf temperatures (Ehleringer *et al.*, 1997), and in drier or more open habitats with higher irradiance and vapour pressure deficits (VPD) (Osborne and Freckleton, 2009; Edwards and Smith, 2010). However, recent research taking advantage of the multiple evolutionary origins of C_4_ photosynthesis in the grass family has demonstrated that ecological and physiological differences are attributable not only to photosynthetic type, but also to differences among lineages (Edwards and Still, 2008; Edwards and Smith, 2010; Taylor *et al.*, 2010; Liu and Osborne, 2015). A key insight is that the outcomes of eco-physiological comparisons between C_3_ and C_4_ grasses in temperate ecosystems are confounded with phylogeny (Edwards *et al.*, 2007). The dominant C_3_ grasses in temperate ecosystems arise from the Pooideae subfamily, which is phylogenetically independent of C_4_ Poaceae and linked with preferences for cooler habitats compared with other C_3_ lineages in the grass family (Edwards and Still, 2008; Edwards and Smith, 2010; Vigeland *et al.*, 2013). Studies that focus on variation in subtropical species and communities are therefore crucial. They have potential to improve our understanding of the ecological factors underpinning the nearly 25% of terrestrial primary productivity contributed by C_4_ grasses (Still *et al.*, 2003), to help predict the impacts of high-yielding C_4_ bioenergy crops (Heaton *et al.*, 2008), and to facilitate attempts to engineer a C_4_ biochemistry into key C_3_ crop species (von Caemmerer *et al.*, 2012).

Because C_4_ photosynthesis fundamentally affects the physiological trade-off between CO_2_ assimilation and water loss through stomata, it has been suggested repeatedly that shifts in plant hydraulics and water use associated with C_4_ evolutionary origins have influenced the ecology of C_4_ species (Edwards and Still, 2008; Osborne and Freckleton, 2009; Edwards and Smith, 2010). Evidence from dicot C_4_ species suggests that increased CO_2_ assimilation relative to water loss facilitates diversification in ecological strategies. C_4_ plants either support greater leaf area for a given stem water supply, or for an equivalent leaf area develop higher-density, lower-conductance stem tissues that are more resistant to hydraulic failure (Kocacinar and Sage, 2003). However, while improvements in the efficiency of CO_2_ assimilation mean that C_4_ photosynthesis can support novel hydraulic strategies, C_4_ photosynthesis in grasses is also associated with the constraint of Kranz anatomy. Kranz anatomy increases the ratio of bundle-sheath/mesophyll tissue (BS:MC) and is linked with decreased inter-vein distances (IVD) (Ueno *et al.*, 2006; Christin *et al.*, 2013; Griffiths *et al.*, 2013; Lundgren *et al.*, 2014). Such leaf-level anatomical differences have been linked with differences in the ecology of C_3_ and C_4_ grass species. In phylogenetically controlled comparisons, the evolution of Kranz anatomy and lower anatomical capacity for stomatal conductance to water (*g*_wmax_) have been shown to match the distribution of C_4_ grasses in drier habitats than C_3_ grasses (Taylor *et al.*, 2012; Griffiths *et al.*, 2013). Unfortunately, studies of leaf hydraulic performance in C_3_ and C_4_ grasses have either compared a limited number of species (Taylor *et al.*, 2018) or, because they were representative of a temperate community, included mostly Pooid C_3_ species that are phylogenetically distant from C_4_ grasses (Ocheltree *et al.*, 2014). Because leaves can contribute as much as 90% of total plant hydraulic resistance (Sack and Holbrook, 2006) and anatomical differences associated with Kranz anatomy may have significant effects on leaf hydraulic properties (Buckley *et al.*, 2015) and construction costs (Niinemets *et al.*, 2007), characterising leaf functional traits associated with hydraulic performance in subtropical grasses should provide key insights to the ecological importance of C_4_ photosynthesis.

Like C_4_ photosynthesis, life history is linked with effects on suites of structural and physiological functional traits (Reich, 2014). It is generally expected that, consistent with the economics of leaf construction and resource use (Grime and Hunt, 1975; Wright and Westoby, 2002), the shorter life spans of annual plants will be linked with lower tissue densities, higher photosynthetic rates, greater allocation to leaf mass and area, and higher relative growth rate than for longer lived perennials (Grime and Hunt, 1975; Garnier *et al.*, 1997). Among herbaceous species, which include the majority of grasses, annual growth strategies are commonly linked with specific adaptations to habitat, for example through escape from drought (Volaire *et al.*, 1998) or competition (Grime, 2006). By improving CO_2_ assimilation efficiency, C_4_ photosynthesis may decrease the relative importance of trade-offs between rapid resource acquisition and resource-use efficiency, and/or support novel ecological strategies linked with changes in growth rate or differential allocation of resources (Long, 1999). There has therefore been ongoing debate about whether C_4_ vegetation is intrinsically more productive (e.g. Osmond *et al.*, 1982; Ehleringer *et al.*, 1997) or exhibits greater niche specialisation (Sage *et al.*, 2011). Recent evidence has supported diversification of functional strategies and expansion by C_4_ populations into a broader range of habitats compared with C_3_ sister groups (Lundgren *et al.*, 2014; Atkinson *et al.*, 2016). Efficiencies associated with C_4_ photosynthesis may, therefore, support diversification in ecological strategies while buffering against the potential costs of constrained leaf anatomy.

In this study, we quantified 26 functional traits for leaves and stems of 42 Poaceae species that grow together in subtropical China, and collected geographic data for climate proxies associated with the global distributions of these species. Our first objective was to assess the relative influences of life history (annual/perennial) and photosynthetic type (C_3_/C_4_) on different functional traits. We expected that (1) annual grasses would show functional traits linked with high-turnover, low-efficiency strategies, for example greater specific leaf areas and water transport capacities but decreased water-use efficiency compared with perennial grasses; and that (2) C_4_ grasses would show high intrinsic water-use efficiency, increased investment in vasculature, and decreased variability in structural properties among species. In addition, because increased resource-use efficiency might compensate for construction costs, we hypothesised that (3) C_4_ photosynthesis would be linked with decreased amounts of functional trait differentiation between annual and perennial species.

Our second objective was to determine whether the habitat preferences of annual and perennial grasses are influenced by photosynthetic type. We expected that differences in habitat characteristics between C_3_ annual and perennial species would be greater than differences between C_4_ annual and perennial species.

## Materials and methods

### Species and growth conditions

**Fig. 1.**
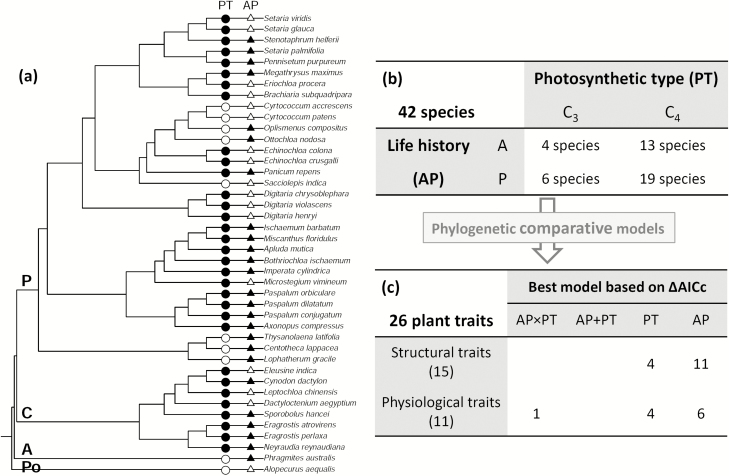
Classification of grass species, data framework, and summary of best-fit models for interspecific variation in functional traits. (a) Cladogram of the 42 grass species from subtropical China. Bold letters near the branch nodes indicate the subfamilies: Panicoideae (P), Chloridoideae (C), Arundinoideae (A), and Pooideae (Po). Symbols indicate the photosynthetic type (PT: C_3_, open circles; C_4_, black circles) and life history (AP: annual, open triangles; perennial, black triangles). (b) Breakdown of species numbers based on categorisations of PT and AP. (c) Breakdown of best-fit models for plant functional traits, indicating the modelled factors and the number of traits for which each model provided the strongest fit based on Table 2.

Experiments were carried out at the South China Botanical Garden, Guangzhou, China (23°11´N, 113°21´E, 100 m altitude). In 2013, 60 native grass species were surveyed widely in different habitats in Guangdong province (mountains, roadsides, farmlands, etc.), transplanted into a greenhouse, and identified at the South China Plants Identification Center, allowing assignment of photosynthetic types according to published literature (Sage *et al.*, 1999; Grass Phylogeny Working Group II, 2012) (see Supplementary Table S1 at *JXB* online). Seeds were harvested in 2013 and plants for experiments were germinated in an incubator in April 2014. After excluding 18 species with low germination rates or that had many similar congeners, 42 species were retained: four C_3_-annuals, six C_3_-perennials, 13 C_4_-annuals, and 19 C_4_-perennials (Fig. 1). This mixture of species was representative of the native flora; in this subtropical monsoon climatic region, there are 316 Poaceae species excluding woody bamboos, of which 9% are C_3_-annuals, 16% C_3_-perennials, 26% C_4_-annuals, and 50% C_4_-perennials (South China Botanical Garden, 2009).

Seedlings were transferred into 4-l pots filled with 1:1 compost (pH 5.6–7, Penglong Gardening Company, Guangdong, China) and sieved topsoil, and were grown in an open-ended, plastic-covered greenhouse until measurements were made in July 2014. The 42 species were replicated in five randomised blocks. All plants were watered daily. During the experimental period, the mean daytime temperature in the greenhouse was 35±3 °C, relative humidity 50±10%, and sunny-day irradiance ~1000 μmol m^–2^ s^–1^, whilst at night the mean temperature was 26±2 °C and relative humidity 96±4%, similar to local ambient conditions (day, 32±4 °C, 73±15%, 1200 μmol m^–2^ s^–1^; night, 28±2 °C, 86±10%) (as determined using an ECH2O Utility, Decagon Devices Inc. WA, USA).

### Functional traits

We determined values for 26 functional traits (Table 1, Supplementary Table S2).

**Table 1.** Abbreviations, units, and classifications for the 26 functional traits and six climatic niche descriptors of the 42 species used in this study.

	Abbreviation and unit	Trait classification
**Plant functional traits**		
Plant height	H (cm)	Structural
Leaf area	LA (cm^2^)	Structural
Leaf thickness	LT (µm)	Structural
Specific leaf area	SLA (cm^2^ g^–1^)	Structural
Leaf dry matter content	LDMC (%)	Structural
Leaf interveinal distance	IVD (µm)	Structural
Maximum diameter of leaf vein bundles	Dlvb (µm)	Structural
Leaf to stem area ratio	*A*_L_/*A*_S_ (cm^2^ mm^–2^)	Structural
Stomatal size	sts (µm^2^)	Structural
Stomatal density	std (mm^–2^)	Structural
Stem cross-section area	SS (mm^2^)	Structural
Diameter of stem vessels	Dsv (µm)	Structural
Stem vessel density	SVD (mm^–2^)	Structural
Stem density	SD (g cm^–3^)	Structural
Leaf carbon content	LC (%)	Structural
Leaf hydraulic conductance	*K*_leaf_ (mmol m^–2^ s^–1^ MPa^–1^)	Physiological
Stem vessel area specific hydraulic conductivity	*K*_S_ (kg m^–1^ s^–1^ MPa^–1^)	Physiological
Leaf specific hydraulic conductivity	*K*_L_ (10^–4^ kg m^–1^ s^–1^ MPa^–1^)	Physiological
Predawn leaf water potential	*Ψ*_pre_ (MPa)	Physiological
Midday leaf water potential	*Ψ*_mid_ (MPa)	Physiological
Leaf turgor loss point	*Ψ*_tlp_ (MPa)	Physiological
Photosynthetic rate	*A* (µmol m^–2^ s^–1^)	Physiological
Stomatal conductance	*g*_s_ (mol m^–2^ s^–1^)	Physiological
Maximum stomatal conductance to water vapour	*g*_wmax_ (mol m^–2^ s^–1^)	Physiological
Intrinsic water use efficiency	WUE_i_ (µmol mol^–1^)	Physiological
Leaf *δ*^13^C	Leaf *δ*^13^C (‰)	Physiological
**Niche descriptors**		
Mean annual precipitation for each species	MAP (mm)	—
Mean annual temperature for each species	MAT (°C)	—
Seasonality of annual precipitation	Ps	—
Seasonality of annual temperature	Ts	—
Mean tree cover in local habitats	Tree cover (%)	—
Mean wet days per year in local habitats	Wet days per year	—

See Supplementary Table S2 for the original data.

Plant height (H) was measured for five mature plants using a ruler. Epidermal peels from both leaf surfaces of three mature fresh leaves per plant were used to determine stomatal traits [guard cell length (gl) and width (gw); stomatal size (sts) and stomatal density (std)]. Because seven of the 42 species were hypostomatous, gl, gw, sts, and std were compared only for the abaxial surface, but values from both surfaces were combined to predict theoretical maximal stomatal conductance to water (*g*_wmax_; see Supplementary Protocol S1). Hand-cut cross-sections from three leaves and stems per plant were used to determine leaf thickness (LT), leaf inter-vein distance (IVD), diameter of major leaf-vein bundles (Dlvb), stem cross-sectional size (SS), stem vessel density (SVD), and mean diameter of stem vessels (Dsv). Measurements were made using an upright microscope (Optec, Chongqing Optec Instrument Co. Ltd, China) equipped with a digital camera and a computerised image analysis system (OPTPro2012 version 4.0, Optec software).

Leaf hydraulic conductance (*K*_leaf_) was measured for the youngest mature leaf per plant, using the high-pressure method (HPM) of Postaire *et al.* (2010) with slight modifications. Leaf blades were excised near the sheath and submerged into a reservoir of degassed and filtered water inside the pressure chamber (Plant Moisture Systems, Corvallis, Oregon, USA), which was used to drive the water through the leaf. *K*_leaf_ was calculated as (Δ*W*_2_−Δ*W*_1_)/[leaf area×time×(*Ψ*_2_−*Ψ*_1_)], where Δ*W*_*i*_ are masses of flow solution collected from the cut surface of the leaf onto pre-weighed tissue papers over 60-s periods. The flow solution was collected first at the balancing pressure for the leaf (*Ψ*_1_), then after a pressure increase of ~0.5 MPa (*Ψ*_2_) (Postaire *et al.*, 2010). Prior to collecting the flow solution, rates of flow were allowed to equilibrate for 5 min at *Ψ*_1_ and *Ψ*_2_. Leaves were scanned and leaf areas were measured using the ImageJ software (Schneider *et al.*, 2012). The total area of the blade was recorded as single leaf area (LA), and the area submerged in the water was determined separately and used to normalise hydraulic conductance. A comparison of the HPM with the evaporative flux method (EFM) (Scoffoni *et al.*, 2016) showed statistically similar results for six grass species, indicating that the HPM provided a reliable estimate of *K*_leaf_ for grass species (see Supplementary Protocol S2, Figs S1, S2, Table S3).

Hydraulic conductivity (*K*_h_) was measured using culm segments with two nodes (~4–30 cm in length depending on species) cut from mature stems and stripped of leaves including sheaths. Culm segments were re-cut underwater, submerged in a tube of degassed and filtered water inside the pressure chamber, then flushed at 0.1 MPa for 5 min to remove air embolisms. Subsequently, the mass of water that flowed through the segments in a 20-s period was determined by collecting the water onto pre-weighed tissues at an initial pressure (*Ψ*_1_, Δ*W*_1_), then after an increase in pressure of ~0.1–0.4 MPa (*Ψ*_2_, Δ*W*_2_). *K*_h_ was calculated as [(Δ*W*_2_–Δ*W*_1_)×stem length]/[time×(*Ψ*_2_−*Ψ*_1_)], and was normalised to stem vessel area (*K*_S_=*K*_h_/*A*_SV_) or total leaf area distal to the segment (*K*_L_=*K*_h_/*A*_L_): *A*_SV_ was calculated as stem cross-section area (*A*_S_)×stem vessel area proportion (VP, see Supplementary Protocol S1), and leaf area (*A*_L_) was determined by scanning. *A*_L_ and *A*_S_ were also used to derive a leaf area to stem cross-section area ratio (*A*_L_/*A*_S_).

Leaves and culms used for hydraulic measurements were dried (65 ^°^C for 72 h) and their masses were determined. Specific leaf area (SLA) for each stem was calculated as the ratio of total leaf area to leaf dry mass. Stem density (SD) was determined as dry mass/volume of the segments, using water displacement to measure volumes. Finally, dried leaves were ground and leaf carbon content (LC) and carbon isotope discrimination (*δ*^13^C) were determined using an isotope-ratio mass spectrometer (Delta V advantage; Thermo Fisher Scientific, MA, USA) at the Chinese Academy of Forestry’s Stable Isotope Laboratory.

Leaf pressure–volume curves were determined using the bench drying method after rehydration (Tyree and Hammel, 1972) (Supplementary Protocol S1). Relationships between *Ψ*_leaf_ and relative water content [(fresh mass−dry mass)/(saturated mass−dry mass)] were analysed to determine the water potential at the turgor-loss point (*Ψ*_tlp_) according to classic models (Schulte and Hinckley, 1985). Dry and saturated masses of leaves were used to determine leaf dry matter content (LDMC) as dry mass/saturated mass.

Net CO_2_ assimilation (*A*) and stomatal conductance to water (*g*_s_), which were used to calculate intrinsic water-use efficiency (WUE_i_=*A*/*g*_s_), were obtained using survey measurements on sunny mornings. Each experimental block of 42 plants took 3 d to measure with an open leaf gas-exchange system (LI-6400XT, LI-COR, Lincoln, NE, USA), which was equipped with a CO_2_ Injector (6400–01) and a Red/Blue LED Light Source (6400-02B). During measurements, photosynthetic photon flux density was 1800 μmol m^−2^ s^−1^, leaf chamber CO_2_ concentration was 380 μmol mol^−1^, and chamber relative humidity 50–70%. The block temperature was not controlled. Measurements were collected after the cuvette had equilibrated for 5 min and values were averaged for two youngest mature leaves from randomly chosen tillers for each plant. Measurements of leaf water potentials from each plant, both pre-dawn (*Ψ*_pre_) and at midday (*Ψ*_mid_), were collected on the same day as gas exchange measurements.

### Niche descriptors

We obtained environmental data using geo-referenced species records from the Global Biodiversity Information Facility (GBIF) collected through GrassPortal (www.grassportal.org). Averages of mean annual temperature (MAT, 1961–1990), mean annual precipitation (MAP, 1961–1990), wet days per year (1961–1990), and tree cover percentage (1992–1993) of habitats were calculated across all geo-referenced localities for each species. Because annuals have distinct growth seasons compared with perennials, we also obtained seasonality data for temperature and precipitation from the WorldClim dataset (http://www.worldclim.org), using *extract* in R (version 3.0.3) (www.r-project.org) package *raster* (Hijmans and Van Etten, 2013).

### Data analysis

We used statistical techniques that control for estimated phylogenetic covariance (Supplementary Protocol S1), because phylogenetic lineage and photosynthetic type act in concert to shape the ecology of the Poaceae (Edwards *et al.*, 2010).

To address coordination among traits and niche descriptors, we carried out a phylogenetic principal component analysis (PPCA) (Felsenstein, 1985) using the *phyl.pca* function in the R package *phytools* (a comparison of PPCA with outcomes of linear discriminant and canonical correlation analyses is provided in Supplementary Protocol S3). Data were log-transformed to fulfil the requirement of normal distribution, and if the original values were negative (*Ψ*_tlp_, *Ψ*_pre_, *Ψ*_mid_, and δ^13^C) absolute values were used. In addition to a pooled analysis we used PPCA to separately analyse 15 ‘structural’ and 11 ‘physiological’ traits. Traits fixed during development were classified as *structural*, e.g. stomatal density and vessel diameter, whereas traits that continuously respond to variation in environmental factors were classed as *physiological*, e.g. stomatal conductance (Table 1).

Because we were interested in contemporary patterns of interspecific trait variation, we modelled comparisons among species mean values using phylogenetic generalised least-squares (*PGLS*; function *pgls* in the R package *caper*). *PGLS* performs well irrespective of the degree of phylogenetic signal, making it ideal for comparisons across large numbers of traits that differ in their associations with phylogeny (Revell, 2010). We used maximum likelihood to estimate Pagel’s *λ* (Pagel, 1999), which assumes a Brownian motion model of trait evolution and which we modelled across a phylogenetic tree extracted from a super tree of Poaceae (Edwards *et al.*, 2010) (Supplementary Protocol S1). For each trait and niche descriptor, and for principal components that explained ≥20% of total variance, we compared four nested linear models: life history (annual/perennial, AP) and photosynthetic type (C_3_ and C_4_ species, PT) were tested independently, additively (AP+PT), and incorporating an interaction (AP×PT).

Because the large number of comparisons and the lack of balance in the number of species in each category (Fig. 1) limited the reliability of *P*-values for model comparisons and post hoc tests, we compared models using an information theoretic framework (Anderson, 2007). To evaluate explanatory power, we used model probability:

$$\begin{array}{l} w_{i}=\frac{exp\left(\text{-}\frac{1}{2}\Delta AICc_{i}\right)}{\sum_{r=1}^{R}exp\left(\text{-}\frac{1}{2}\Delta AICc_{r}\right)} \end{array}$$

ΔAICc are differences in corrected Akaike information criterion scores (AICc), between alternative models (AP×PT, AP+PT, PT, and AP), that use the model with the minimum AICc as a reference (Anderson, 2007). The numerator is equivalent to the likelihood of the model of interest (model *i*), and the denominator is the sum of likelihoods for all R (=4) models. Model probabilities were compared using evidence ratios (*w*_i_/*w*_j_), where *w*_i_ is the probability of the focal model and *w*_j_ is the probability of a comparator model. Higher evidence ratios indicate greater relative support for focal models, and comparisons between the best model (minimum AICc) and the second-best model (the second-lowest AICc) are indicated specifically by *w*_min_/*w*_2_ (Table 2).

**Table 2.** Comparisons of phylogenetic generalised least-squares (*PGLS*) models that estimate the effects of photosynthetic type (PT; C_3_ and C_4_) and/or life history (AP; annual and perennial) on (a) plant functional traits (*n*=42 species), (b) principal component scores for trait variation, (c) niche descriptors (*n*=34 species, due to the lack of climate data for eight species), and (d) principal component scores for niche variation.

	^1^ΔAICc				^2^Model probability (*w*_i_)				^3^Evidence Ratio (*w*_min_/*w*_2_)	Best model	*λ* for best model
	AP×PT	AP+PT	PT	AP	AP×PT	AP+PT	PT	AP			
**(a) Plant functional traits**											
IVD (µm)	6.89	3.39	**0.00**	14.05	0.026	0.151	**0.822**	0.001	5.45	PT	0.00
SD (g cm^–3^)	6.51	3.38	4.97	**0.00**	0.030	0.141	0.064	**0.765**	5.42	AP	0.00
–*Ψ*_tlp_ (MPa)	6.90	3.38	13.20	**0.00**	0.026	0.152	0.001	**0.821**	5.42	AP	0.00
Leaf *δ*^13^C (‰)	5.62	3.35	**0.00**	109.00	0.048	0.150	**0.802**	0.000	5.34	PT	0.62
*K*_leaf_ (mmol m^–2^ s^–1^ MPa^–1^)	**0.00**	5.40	3.34	4.07	**0.721**	0.048	0.136	0.094	5.31	AP×PT	0.24
–*Ψ*_pre_ (MPa)	5.95	3.31	**0.00**	6.32	0.040	0.149	**0.778**	0.033	5.23	PT	0.00
SLA (cm^2^ g^–1^)	6.72	3.28	13.32	**0.00**	0.028	0.158	0.001	**0.813**	5.16	AP	0.52
Dsv (µm)	6.33	**3.14**	3.88	**0.00**	0.030	0.149	0.103	**0.717**	4.81	AP	0.00
*g*_s_ (mol m^–2^ s^–1^)	6.37	**2.86**	**2.87**	**0.00**	0.027	0.158	0.157	**0.658**	4.18	AP	0.36
Dlvb (µm)	4.84	**2.74**	4.60	**0.00**	0.062	0.176	0.069	**0.693**	3.94	AP	0.00
sts (µm^2^)	5.06	3.37	**0.00**	**2.70**	0.052	0.122	**0.656**	0.170	3.86	PT	0.20
H (cm)	5.03	**2.30**	7.09	**0.00**	0.057	0.222	0.020	**0.701**	3.16	AP	0.00
LDMC (%)	3.25	**2.25**	11.15	**0.00**	0.129	0.213	0.002	**0.656**	3.08	AP	0.08
*A*_L_/*A*_S_ (cm^2^ mm^–2^)	5.27	**2.09**	8.89	**0.00**	0.050	0.245	0.008	**0.697**	2.84	AP	0.00
LT (µm)	6.26	3.36	**2.02**	**0.00**	0.027	0.117	0.228	**0.627**	2.75	AP	0.00
*g*_*wmax*_ (mol m^–2^ s^–1^)	5.43	**2.77**	**1.76**	**0.00**	0.038	0.145	0.240	0.578	2.41	AP	0.57
LA (cm^2^)	**2.98**	**1.69**	6.63	**0.00**	0.133	0.254	0.021	0.591	2.33	AP	0.17
SS (mm^2^)	**1.48**	**1.16**	4.26	**0.00**	0.221	0.260	0.055	0.464	1.79	AP	0.00
WUE_i_ (µmol mol^–1^)	4.35	**0.80**	**0.00**	28.38	0.064	0.376	0.561	0.000	1.49	PT	0.45
LC (%)	6.87	**3.38**	**0.00**	**0.69**	0.017	0.096	0.519	0.368	1.41	PT	0.00
std (mm^–2^)	**0.68**	**1.96**	**0.00**	**1.06**	0.266	0.140	0.374	0.220	1.40	PT	0.08
*K*_S_ (kg m^–1^ s^–1^ MPa^–1^)	5.80	**3.02**	**0.68**	**0.00**	0.028	0.111	0.358	0.503	1.40	AP	0.00
*A* (µmol m^–2^ s^–1^)	4.13	**0.59**	**0.00**	26.72	0.068	0.398	0.534	0.000	1.34	PT	0.00
–*Ψ*_mid_ (MPa)	4.61	**3.17**	**0.36**	**0.00**	0.047	0.096	0.390	0.467	1.20	AP	0.00
SVD (mm^–2^)	**0.10**	**1.80**	**2.43**	**0.00**	0.358	0.153	0.112	0.377	1.05	AP	0.00
*K*_L_ (10^–4^ kg m^–1^ s^–1^ MPa^–1^)	6.35	3.31	**0.07**	**0.00**	0.019	0.087	0.439	0.455	1.04	AP	0.51
**(b) Principal component scores for trait variation**											
PC2 of physiological traits	6.33	3.39	8.41	**0.00**	0.034	0.148	0.012	**0.806**	5.45	AP	0.44
PC1 of physiological traits	**3.08**	**2.04**	**0.00**	62.47	0.136	0.229	**0.635**	0.000	2.77	PT	0.00
PC1 of structural traits	**1.84**	**1.91**	14.53	**0.00**	0.223	0.216	0.000	0.561	2.51	AP	0.00
PC1 of all traits	**0.00**	**0.18**	13.14	**2.04**	0.439	0.402	0.001	0.158	1.09	AP×PT	0.00
**(c) Niche descriptors**											
Precipitation seasonality	6.61	**3.15**	**2.69**	**0.00**	0.024	0.138	0.173	**0.665**	3.84	AP	0.50
Temperature seasonality	6.03	**2.35**	5.45	**0.00**	0.034	0.217	0.046	**0.703**	3.24	AP	0.00
MAP (mm)	5.74	**2.14**	**2.91**	**0.00**	0.035	0.210	0.143	**0.612**	2.92	AP	0.00
Wet days per year	**2.71**	**0.00**	**1.86**	**2.14**	0.129	0.501	0.198	0.172	2.53	AP+PT	0.00
MAT (°C)	7.01	3.39	**1.82**	**0.00**	0.019	0.114	0.249	**0.619**	2.48	AP	0.88
Tree cover (%)	4.24	**0.70**	**1.57**	**0.00**	0.053	0.309	0.200	0.438	1.42	AP	0.00
**(d) Principal component scores for niche variation**											
PC1 of six niche descriptors	6.10	**2.81**	4.94	**0.00**	0.034	0.178	0.061	**0.726**	4.08	AP	0.00
PC2 of six niche descriptors	6.70	**3.05**	**0.00**	**2.58**	0.023	0.143	**0.654**	0.180	3.63	PT	0.66

Traits and principal components are ranked by the power to identify a single ‘best’ model (evidence ratio, *w*_min_/*w*_2_) and dashed lines separate models at evidence ratio >5 and >2.5. Models are compared using differences in the corrected Akaike Information Criterion (ΔAICc) and their probability within the four models (*w*_*i*_).

^1^ Bold type highlights models with ΔAICc<3.22 (*w*_min_/*w*_2_*≈*5.00).

^2^ Bold type highlights models with *w*_i_>0.60.

^3^ Among the four *w*_i_ values, the highest, which is associated with the minimum AICc, is defined as *w*_min_, the second highest is used as *w*_2_.

## Results

### Impact of life history and photosynthetic type on functional traits

**Fig. 2.**
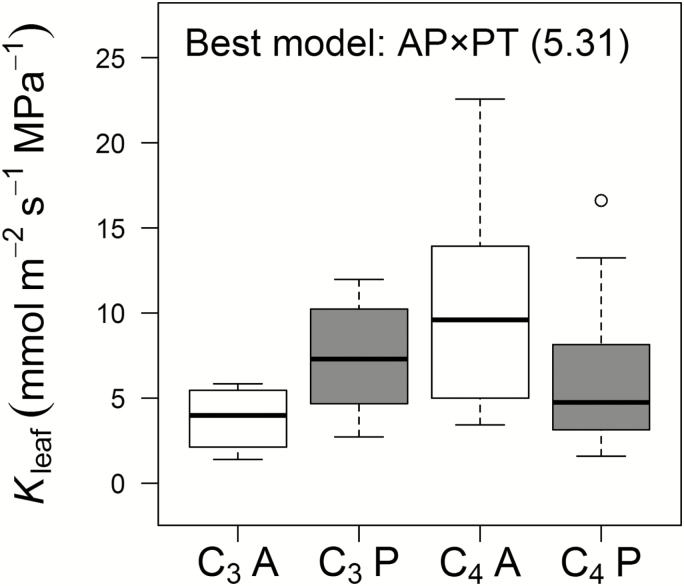
Interaction between life history (AP: annual, open boxes; perennial, grey boxes) and photosynthetic type (PT: C_3_, left; C_4_, right) affecting leaf hydraulic conductance (*K*_leaf_) of the 42 grass species. The box-plots show quartiles for each trait with extreme values as circles. Sample sizes for C_3_-A, C_3_-P, C_4_-A, and C_4_-P were 4, 6, 13 and 19, respectively. The best-fit model and its evidence ratio are shown.

Of the 26 traits, seven (*K*_leaf_, SD, *Ψ*_tlp_, SLA, IVD, *δ*^13^C, and *Ψ*_pre_) showed support for ‘best’ models with *w*_min_/*w*_2_>5 (Table 2a). Of these, *K*_leaf_ was best modelled by AP×PT, with the highest values from C_4_ annuals and the lowest from C_3_ annuals, whilst perennials of both photosynthetic types had similar, intermediate values (Fig. 2). SD, *Ψ*_tlp_, and SLA were all clearly determined by AP; and IVD, δ^13^C, and *Ψ*_pre_ depended on PT (Table 2a). Best-fitting models for a further eight traits showed evidence ratios in the range 2.5–5. Of these, seven traits (Dsv, *g*_s_, Dlvb, H, LDMC, *A*_L_/*A*_S_, LT) were best modelled as dependent on AP, and one, sts, was best modelled as depending on PT (Table 2a). In combination, traits for which best-fit models had *w*_min_/*w*_2_>2.5 showed the following characteristics. Annuals were shorter and had higher *g*_s_. Their leaves were thinner, with smaller vascular bundles (Dlvb), higher SLA, lower LDMC, and less negative *Ψ*_tlp_ than perennials. The stems of annuals were less dense (SD), with narrower vessels (Dsv), and supported relatively small leaf areas (*A*_L_/*A*_S_) (Table 3a). Meanwhile, C_4_ species showed smaller IVD and sts, and less negative *δ*^13^C and *Ψ*_pre_ than C_3_ species (Table 3b).

**Table 3.** Plant functional traits for which (a) life history or (b) photosynthetic type is supported as the sole explanatory factor with an evidence ratio >2.5.

(a)	Annual (17 species)	Perennial (25 species)
**SD (g cm** ^**–3**^)	122.7±11.08	144.3±12.04
***Ψ***_**tlp**_ **(MPa)**	–1.2±0.04	–1.5±0.04
**SLA (cm** ^**2**^ **g** ^**–1**^)	425.4±19.43	321.7±16.56
Dsv (µm)	22.6±1.22	28.1±1.92
*g*_s_ (mol m^–2^ s^–1^)	0.5±0.05	0.4±0.03
Dlvb (µm)	70.9±6.86	92.4±6.94
H (cm)	48.2±4.91	100.2±13.49
LDMC (%)	20.4±0.66	25.4±0.95
*A*_L_/*A*_S_ (cm^2^ mm^–2^)	23.6±3.24	43.5±5.05
LT (µm)	139.0±5.41	157.8±8.33
(b)	C_3_ (10 species)	C_4_ (32 species)
**IVD (µm)**	314.8±44.68	173.4±7.52
**Leaf *δ*** ^**13**^ **C (‰)**	–29.6±0.23	–13.9±0.15
***Ψ***_**pre**_ **(MPa)**	–0.09±0.012	–0.07±0.004
sts (µm^2^)	209.1±24.89	156.6±9.72

Data are means (±s.e.m.), and bold text highlights traits with evidence ratios >5.

**Fig. 3.**
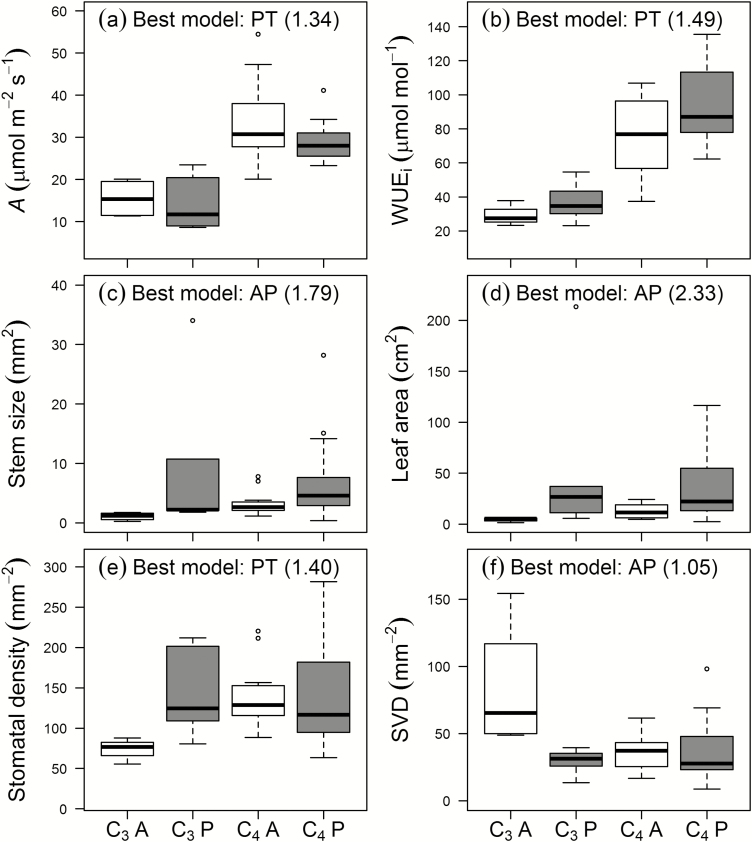
Functional traits for which AP×PT or AP+PT had similar explanatory powers compared with the best-fitting single-factor models (AP: annual, open boxes; perennial, grey boxes; or PT: C_3_, left; C_4_, right). The box-plots show quartiles for each trait with extreme values as circles. Sample sizes for C_3_-A, C_3_-P, C_4_-A, and C_4_-P were 4, 6, 13 and 19, respectively. The best-fit models and evidence ratios are shown.

Consistent with the moderate support for many of the best models, there was also support for AP+PT models for all traits (ΔAICc ≤3.39; *w*_min_/*w*_AP+PT_ ≤5.45) except *K*_leaf_ (*w*_AP×PT_/*w*_AP+PT_=5) (Table 3). The greatest statistical support for AP+PT effects was found for *A* (*w*_AP+PT_=0.4), WUE_i_ (*w*_AP+PT_=0.38), SS (*w*_AP+PT_=0.26), and LA (*w*_AP+PT_=0.25; Fig 3a–d). For these four traits, the ‘best’ models were single-factor (AP or PT) but evidence ratios for these were relatively low (*w*_min_/*w*_2_≤2.33). For *A* and WUE_i_ the best model was PT, and while C_4_ species had greater *A* and WUE_i_, *A* was slightly higher and WUE_i_ lower within annuals than within perennials (Fig. 3a, b). For SS and LA, AP was the best model; annuals were clearly much smaller, and both leaves and stems tended to be smaller within C_3_ species (Fig. 3c, d).

Importantly, in addition to strong evidence for AP×PT affecting *K*_leaf_, AP×PT models fitted better than AP+PT models for two traits: std (*w*_AP×PT_/*w*_AP+PT_=1.9) and SVD (*w*_AP×PT_/*w*_AP+PT_=2.34). These two traits had single-factor best models with evidence ratios (*w*_min_/*w*_2_) ≤1.4 (Table 2: std, PT; SVD, AP), and were similar among C_3_-perennials, C_4_-annuals, and C_4_-perennials, but C_3_-annuals showed lower std (Fig. 3e) and higher SVD (Fig. 3f).

Of the remaining traits, *g*_wmax_, *Ψ*_mid_, *K*_S_, and *K*_L_ were explained best by AP, but without a clear difference in explanatory power compared with PT, and the best model for LC was PT, but AP had similar explanatory power (Table 2a). For these five traits, lower evidence ratios for best-fitting, single-factor models and lack of support for AP+PT or AP×PT as secondary models (that would explain the low power of the primary models) meant that there was no convincing evidence for a strong fit by any of the four alternative models (Supplementary Fig. S3).

In summary, the majority of traits were most clearly linked with life history; however, in addition to expected contrasts between C_3_ and C_4_ photosynthetic types our data showed that differences between annual and perennial grasses in *K*_leaf_, SVD, and std depended on photosynthetic type.

### Impact of life history and photosynthetic type on trait coordination: PPCA

**Fig. 4.**
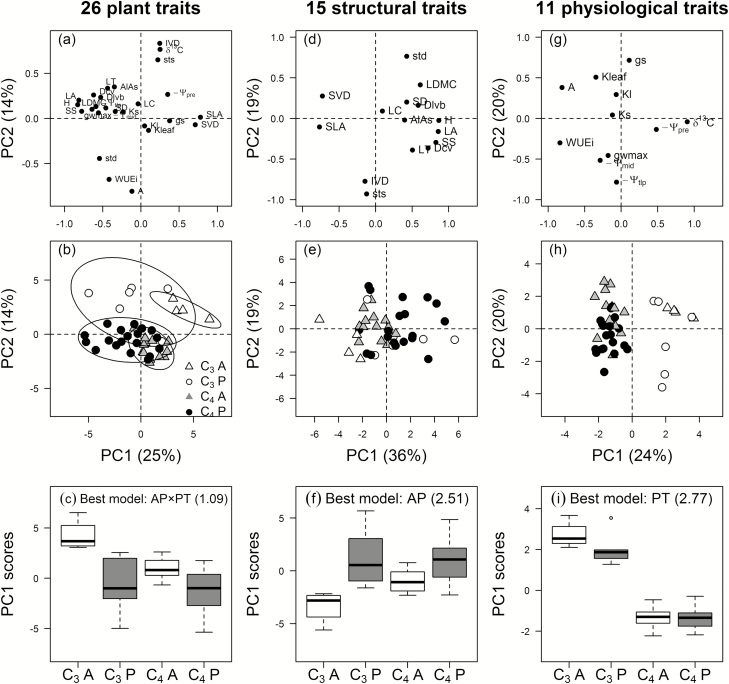
Phylogenetic principal component analysis (PPCA) for the 26 functional traits (a–c) of 42 C_3_ and C_4_ grasses with annual or perennial life histories. Traits were secondarily classified as (d–f) 15 structural traits or (g–i) 11 physiological traits, with the first two principal components (PCs) and species scores reported and analysed. (a, d, g) PC loadings of the 26 traits; (b, e, h) scores for 42 species in four groups: C_3_-A, C_3_ annuals; C_3_-P, C_3_ perennials; C_4_-A, C_4_ annuals; C_4_-P, C_4_ perennials; and (c, f, i) box-plots of PC1 species scores. Percentages of variance explained by PCs are shown in the axis labels. Trait abbreviations are given in Table 1.

For the full set of 26 functional traits, the first two PCs explained 25% and 14% of total variation, respectively (Fig. 4a, b). PC1 separated annuals and perennials, whereas PC2 separated C_3_ and C_4_ species. Separation of AP along PC1 was consistent with differences between larger perennial species (H, LA, and SS; negative association) with low SLA and SVD, and smaller annual species with higher SLA and SVD. Importantly, although PC1 scores for annuals were higher than for perennials within both photosynthetic types, C_3_-perennials, C_4_-annuals, and C_4_-perennials showed similar scores, while C_3_-annuals were clearly distinguished from the other three groups by higher scores (Fig. 4c). This pattern was supported by the best-fitting AP×PT model for PC1, and although an AP+PT model could explain PC1 almost as well as AP×PT (ΔAICc=0.18, *w*_min_/*w*_2_=1.09), PT or AP alone were much poorer models for PC1 (*w*_i_/*w*_j_>2.5; Table 2b). Although models were not compared because of the relatively small proportion of variance explained, PC2 for the full set of functional traits clearly separated species on the basis of PT (Fig. 4b): greater IVD and sts, combined with more negative *δ*^13^C (C_3_ traits), were separated from higher *A*, higher WUE_i_, and greater std (C_4_ traits; Fig. 4a).

PPCA of the 15 structural traits explained 36% and 19% of variation with the first two PCs (Fig. 4d, e). Scores for SLA, SVD, and size-related traits again showed strong opposite associations along PC1, separating annuals and perennials (Fig. 4d–f). Graphically, this life history axis was, again, linked with greater separation in structure between annual and perennial C_3_ species compared with their C_4_ relatives (Fig 4f; Table 2b). However, although there was some support for AP+PT and AP×PT as alternative models with similar power (ΔAICc≤1.91, *w*_i_~0.22; Table 2b) the best model for PC1 was AP alone (*w*_min_/*w*_2_=2.5; Table 2b). Although PC2 for the structural traits explained <20% of total variance and was not modelled, it was most strongly associated with IVD (negative scores) and a trade-off between sts (negative scores) and std (positive scores). It was therefore surprising that C_3_ and C_4_ species did not separate along PC2 (Fig. 4e, f).

Among the 11 physiological traits, the first two PCs explained less variation but PC2 was slightly more important than for structural traits (PC1, 24%; PC2, 20%; Fig. 4g–i). In contrast with structural traits, PC1 for physiological traits clearly distinguished C_3_ and C_4_ species (*w*_i_=0.635; Table 2b). C_4_ grasses had hi*g*her *A* and WUE_i_, and C_3_ grasses greater IVD and more negative δ^13^C (Fig. 4g–i). As for structural traits, there was moderate support for the primary model of PC1 that separated species by PT (*w*_min_/*w*_2_=2.77), associated with greater differences in score between annual and perennial C_3_ species (Fig. 4i). However, unlike PC1 for the structural traits, the AP+PT model (*w*_i_=0.229) was clearly a better secondary fit to scores along PC1 than the AP×PT model (*w*_i_=0.136; Table 2b). Along PC2, AP was strongly supported as the best-fitting model (ΔAICc≥3.39; *w*_min_/*w*_2_=5.45; Fig. 4h; Table 2b). Annual species had higher *A*, *g*_s,_ and *K*_leaf_, together with less negative *Ψ*_tlp_ and *Ψ*_mid_, and, surprisingly, lower *g*_wmax_ (Fig. 3g).

In summary, PPCA analysis showed that variation in structural traits was strongly aligned with differences in life history, and that even among physiological traits almost as much variation was explained by life history (20%, PC2) as by photosynthetic type (24%, PC1). Importantly, when all 26 traits were considered, differentiation of annual and perennial species along the primary axis of variation depended on photosynthetic type: there were greater differences between annual and perennial C_3_ species than between annual and perennial C_4_ species.

### Impact of life history and photosynthetic type on niche descriptors

**Fig. 5.**
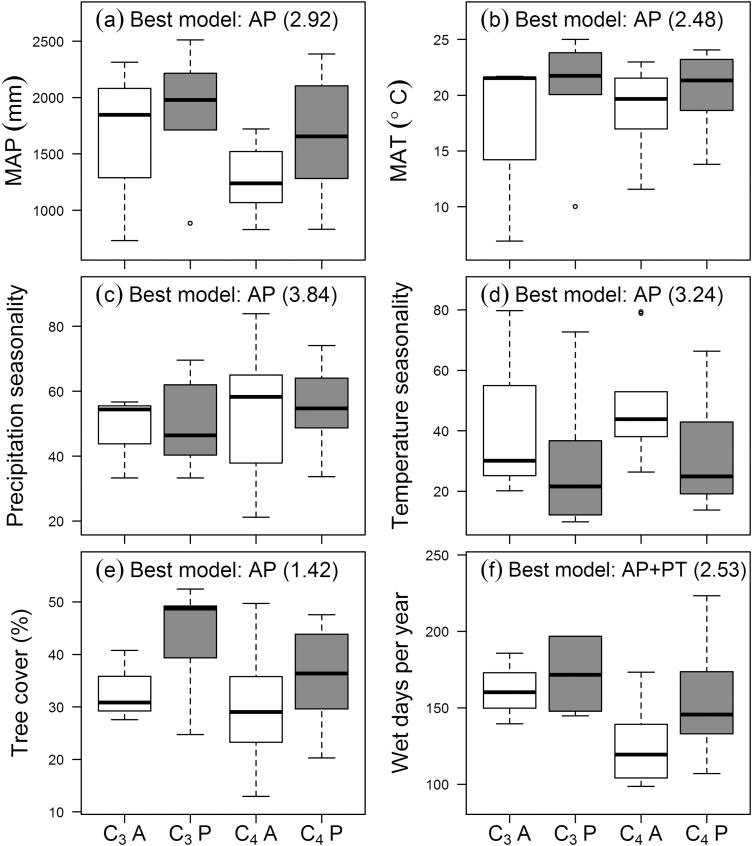
Niche descriptors for 34 grass species sampled in subtropical China, grouped by life history (AP: annual, open boxes; perennial, grey boxes) and photosynthetic type (PT: C_3_, left; C_4_, right). The box-plots show quartiles for each trait with extreme values as circles. Sample sizes for C_3_-A, C_3_-P, C_4_-A, and C_4_-P were 3, 6, 9 and 16, respectively. The best-fit models and evidence ratios are shown. MAP, mean annual precipitation; MAT, mean annual temperature; seasonality is the coefficient of variation of monthly values. Only 34 of the 42 study species were included in this analysis because climatic data were not available for the other eight.

Tree cover, MAT, MAP, and seasonality of temperature and precipitation were all best explained by AP, with support increasing respectively from weak to moderate (*w*_min_/*w*_2_: 1.42–3.84; Table 2c). In both photosynthetic types, annual species were linked with lower MAP and MAT, decreased tree cover, and increased seasonality of temperature and precipitation (although differences were weaker for seasonality of precipitation; Fig. 5a–e). AP×PT was a weak model of all niche descriptors (*w*_i_≤0.129), but AP+PT received moderate support as the best model for wet days per year (*w*_i_=0.5; *w*_min_/*w*_2_=2.53), and was ranked second for tree cover (*w*_i_=0.31), MAP (*w*_i_=0.21), and temperature seasonality (*w*_i_=0.22) (Table 2c). Greater numbers of wet days were characteristic of habitats for both C_3_ and perennial species (Fig. 5f) and, in addition to differences between annuals and perennials, C_3_ grasses were associated with greater tree cover and MAP, and lower temperature seasonality. Moderate support for PT as an alternative model for tree cover (*w*_i_=0.2) was due to slightly higher mean values among C_3_ species (Fig. 5).

### Characterisation of niche spaces: PPCA

**Fig. 6.**
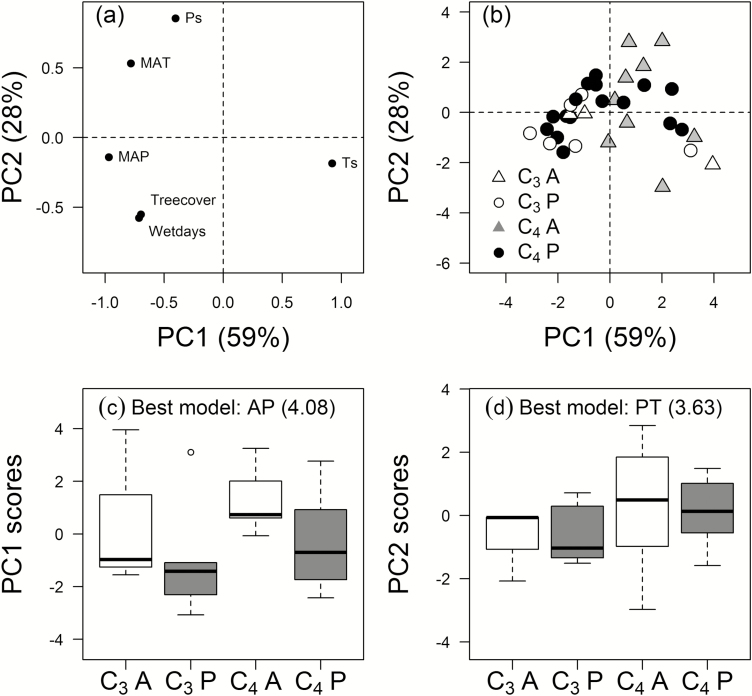
Phylogenetic principal component analysis (PPCA) for six niche descriptors of 42 C_3_ and C_4_ grasses with annual or perennial life histories. (a) Principal component (PC) loadings of the six niche descriptors; (b) scores for 42 species in four groups: C_3_-A, C_3_ annuals; C_3_-P, C_3_ perennials; C_4_-A, C_4_ annuals; C_4_-P, C_4_ perennials; and (c, d) box-plots of PC1 and PC2 species scores. Percentages of variance explained by PCs are shown in the axis labels. Trait abbreviations are given in Table 1.

The six niche descriptors were far more effectively summarised by PPCA than were the larger array of functional traits: the first two PCs explained 87% of total variation in niche descriptors (PC1, 59%; PC2, 28%; Fig. 6a, b). PC1 was best characterised (*w*_min_/*w*_2_=4.08, *w*_i_=0.73; Table 2) as separating annual and perennial species by seasonality of temperature, MAP, and MAT (Fig. 6a): annual species were associated with increased seasonality of temperature, and decreased MAP and MAT. PC2 was best modelled as dependent on PT (*w*_min_/*w*_2_=3.63, *w*_i_=0.65; Table 2d) with a relatively large correction for phylogenetic covariance (*λ*=0.66). Along PC2, C_4_ species were associated with greater MAT and seasonality of precipitation, and decreased tree cover and wet days per year (Fig. 6b–d).

### Influence of phylogeny on trait distributions

For most of the 26 functional traits and four associated PCs, phylogenetic signal had weak effects on the best-fitting models (*λ*≤0.2 for 22 of the 30 models; Table 2a, b). The highest *λ* value for a best-fitting model was 0.62 for δ^13^C when accounting for PT (Table 2; *λ* for all models are provided in Supplementary Table S4 ).

For the individual niche descriptors, both seasonality of precipitation (*λ*=0.50) and MAT (*λ*=0.88) showed moderate to strong phylogenetic signals, in each case after accounting for PT as the best-fitting model (other niche descriptors showed *λ≈*0 for their best-fitting models; Table 2). Consistent with these results, PC2 for the niche descriptors, which was defined by a contrast between greater seasonality of precipitation and MAT versus greater tree cover and numbers of wet days, also showed a phylogenetic signal after accounting for PT as a primary effect (*λ*=0.66, Table 2).

## Discussion

The majority of variation in the 26 functional traits and six niche descriptors of the grasses we studied was best characterised by differences in life history: at the local scale, therefore, life history is a key factor shaping the functional ecology of subtropical grasses, and is more important for trait differentiation than photosynthetic type. Annual grasses were smaller, with higher SLA, denser stems, less-negative *Ψ*_tlp_, and higher *g*_s_. Photosynthetic type had important effects on physiological traits and affected how several traits linked with hydraulic function differed between annual and perennial species. *K*_leaf_, a trait at the nexus of leaf structure and function (Sack and Holbrook, 2006), was lower in C_3_ annuals and higher in C_4_ annuals than in perennial species of either photosynthetic type. C_3_-annuals also had greater stem vessel and lower stomatal densities than C_4_ annuals or perennials of both photosynthetic types. We found no strong evidence for interactions between annual/perennial life history (AP) and photosynthetic type (PT) affecting niche descriptors. Annual grasses had clear preferences for drier, less shaded, and more seasonal habitats, and C_4_ species, especially annuals, were found to inhabit locations with higher temperatures, low tree cover, and lower, more variable rainfall.

### Interplay between life history and photosynthetic type

High-turnover, resource-inefficient annual strategies are likely to be economic only where resources are sufficiently limited that larger, more competitive plants capable of greater resource capture are excluded (Grime and Hunt, 1975; Reich, 2014). Compatible with this, we found that annuals exploited drier habitats with less tree cover that were more seasonal and cooler on an annual basis than those of their perennial relatives.

Notably, of the four classes of grass species we investigated, C_4_-annuals tended to inhabit the driest locations, and our findings suggest that C_3_ and C_4_ annuals differ in key traits that may influence resistance to loss of hydraulic function. C_3_-annuals were characterised by low stomatal densities, high stem vessel densities, and low *K*_leaf_, while C_4_ annuals showed higher *K*_leaf_ compared with perennial species. Moreover, because differences in *g*_s_ were relatively small, *K*_leaf_/*g*_s_ (supply versus demand) was also greatest in C_4_-annuals and smallest in C_3_-annuals. Higher *K*_leaf_/*g*_s_ should decrease stomatal sensitivity to VPD (Brodribb and Holbrook, 2003; Osborne and Sack, 2012; Ocheltree *et al.*, 2014), so this result supports greater impacts of drought and high VPD conditions on gas exchange of C_3_ than C_4_ annuals. Meanwhile, the high stem vessel densities, low stomatal densities, and low *K*_leaf_ of C_3_ annuals further suggest a hydraulic system tuned to combat hydraulic failure by minimising vulnerability. We were, however, surprised to find that C_3_ and C_4_ perennials showed substantial overlap in *K*_leaf_.

In C_4_ plants, smaller IVD is linked with improved photosynthetic efficiency, especially quantum yield (Ogle, 2003), and might increase *K*_leaf_ relative to demand from transpiration (Osborne and Sack, 2012). For annual species, differences in *K*_leaf_ were consistent with smaller IVD in C_4_ species resulting in higher *K*_leaf_. However, consistent with a study that compared temperate species (Ocheltree *et al.*, 2014), C_3_ and C_4_ perennials in our study had similar *K*_leaf_. Going forwards, it will be important to determine the consequences for leaf hydraulic function of differences among grass lineages (Liu and Osborne, 2015), of structural differences that underpin leaf size and thickness, of lateral vessels that affect vein length per area (Ueno *et al.*, 2006; Sack and Scoffoni, 2013), and of bundle-sheath tissues (Griffiths *et al.*, 2013).

### Independent effects of life history and photosynthetic type

Life history was the single factor that best explained functional trait variation in our experiment. As expected, annual grasses tended to be shorter, with thinner leaves and stems (Garnier *et al.*, 1997) and low *A*_L_/*A*_S_. The thinner leaves of annuals also had smaller vascular bundles and tended to show higher *g*_s_, lower WUE_i_, and less-negative *Ψ*_tlp_. These traits, in particular higher *g*_s_ and lower WUE_i_, support our hypothesis of high-demand hydraulic systems in annuals. Less-negative *Ψ*_tlp_ suggests a tendency towards greater vulnerability of leaf performance to declining water status, but may also be associated with rapid leaf wilting that might counteract lower WUE_i_ in high-irradiance environments. Wilting can improve leaf level WUE by decreasing interception of irradiance, and hence transpiration (Turner and Begg, 1981).

Expected differences between C_3_ and C_4_ grasses were clearly represented in our dataset. Along with less-negative *δ*^13^C (Farquhar *et al.*, 1989) and smaller IVD (Sage, 2004; Ueno *et al.*, 2006; Lundgren *et al.*, 2014), C_4_ grasses had higher *A* and WUE_i_ (Pearcy and Ehleringer, 1984; Taylor *et al.*, 2010;, 2011). However, some of our results are at variance with those of previous studies. For example, previous studies of eudicots have shown that *K*_S_ and/or *K*_L_ can be lower among C_4_ species, which may reduce vulnerability to hydraulic failure (Kocacinar and Sage, 2004). We found no good evidence for differences in *K*_S_ or *K*_L_ based on photosynthetic type. In our results, AP was marginally better than PT as an explanation for differences in *K*_S_ and *K*_L_, but there was little support for systematic differences due to AP or PT in either of these traits. In addition, while we confirmed that smaller, more closely spaced stomata were broadly characteristic of C_4_ species, this was not associated with a shift in *g*_wmax_, as has been reported previously (Taylor *et al.*, 2012). Two features of our study might explain the lack of a PT effect on *g*_wmax_. First, the C_4_ genus *Aristida* was not represented in the subtropical grass flora we studied; species of *Aristida* are characteristic of dry habitats and were a key group showing low *g*_wmax_ in Taylor *et al.* (2012). Second, our experimental design increased the representation of annual species compared with previous studies, and we found that low std was a particular feature of C_3_ annuals. This may explain why AP was a marginally better explanation of *g*_wmax_ than PT. Previous evidence has suggested links between *g*_wmax_ and habitat water availability (Taylor *et al.*, 2012), so lower *g*_wmax_ within C_3_ annuals in this subtropical environment is consistent with the other lines of evidence from our experiment that suggest they are commonly exposed to water stress.

### Niche descriptors of subtropical grasses

For all of the niche descriptors there was some evidence that PT had independent effects on species niche preferences: after accounting for AP differences, the ranges of C_4_ species extended into drier, more seasonal locations than those of C_3_ species. Our results therefore supported the broad hypothesis that C_4_ photosynthesis often provides advantages in drier, more open habitats (Osborne and Freckleton, 2009; Edwards and Smith, 2010). By contrast, we found that AP was always a stronger explanation for temperature preferences than PT: annuals were linked with lower average MAT and increased seasonality of temperature. Preferences for MAT were also linked with the strongest phylogenetic signals in our dataset, consistent with previous studies (Edwards and Still, 2008; Edwards and Smith, 2010; Liu and Osborne, 2015). Our results therefore support previous suggestions that thermal constraints are less important than tree cover and rainfall in determining C_3_/C_4_ distributions in the subtropics (Edwards and Still, 2008; Edwards and Smith, 2010). However, we chose to investigate a subtropical community to avoid strong effects of deeper divergences and global diversity within the Poaceae that affect studies in temperate communities (Edwards and Still, 2008; Edwards and Smith, 2010). As a consequence, the C_3_ subfamilies Pooideae and Arundinoideae were each represented by a single species, and the subfamilies, Bambusoideae, Ehrhartoideae, Aristidoideae, Micrairoideae, and Danthonioideae were not represented at all. Thus, the relatively small impact of phylogeny compared with life history and photosynthetic type that we observed is likely to be particular to the subtropical species assemblage. Impacts of phylogeny will be greater when comparisons are made in communities that include species with diverse climate preferences, or when phylogenetic diversity among Poaceae is more broadly represented.

## Conclusions

For the subtropical grass species that we studied, life history was the predominant explanation of differences in most functional traits and niche descriptors. As we expected, annual grasses showed functional traits related with high-turnover and low-efficiency strategies. Annual grasses, in particular C_4_-annuals, also tended to be distributed in drier and more seasonal habitats than perennial grasses. A particularly novel finding was that functional trait contrasts between annual and perennial species interacted with photosynthetic type. Specifically, trait variation between annual and perennial grasses was greater among C_3_ than C_4_ species. Hydraulic traits, in particular *K*_leaf_, were central to this finding. These results suggest that interactions with life history are a key factor to be considered when trying to establish the impacts of photosynthetic type or phylogeny on species functional ecology.

## Supplementary Data

Supplementary data are available at *JXB* online.

Protocol S1. Supplementary methods for determining functional traits.

Protocol S2. Comparison of the evaporative flux method and high-pressure method for determining *K*_leaf_.

Protocol S3. Comparison of phylogenetic principal component analysis with linear discriminant analysis and canonical correlation analysis for data in this study.

Table S1. Phylogenetic clades, species names, and groups of the 42 species used in this study.

Table S2. Values for the 26 functional traits and six climatic niche descriptors of the 42 species used in this study.

Table S3. Raw data used to compare the evaporative flux method and high-pressure method for determining *K*_leaf_.

Table S4. Pagel’s *λ* for phylogenetic generalised least-squares models to analyse the effects of photosynthetic type and life history on plant traits, principal component scores, and niche descriptors.

Fig. S1. Comparisons between the evaporative flux method and high-pressure method to determine *K*_leaf_.

Fig. S2. Images of leaf cross-sections of four typical species used in this study to determine *K*_leaf_.

Fig. S3. Five functional traits for which the photosynthetic type and life history models had similar explanatory power.

Supplementary Data

Supplementary Table S2 and S3
