# Supplementary material for: Life history is a key factor explaining functional trait diversity among subtropical grasses, and its influence differs between C3 and C4 species
Source: J Exp Bot. 2019 Feb 11;70(5):1567–80. doi: 10.1093/jxb/ery462 (PMC6411383; doi:10.1093/jxb/ery462)
Supplement: Supplementary Data [file ery462_suppl_supplementary_data.pdf]

Supplementary Data

Life history is a key factor explaining functional trait diversity among subtropical grasses, and its influence differs between C_3 and C_4 species

Hui Liu, Samuel H. Taylor, Qiuyuan Xu, Yixue Lin, Hao Hou, Guilin Wu, Qing Ye

Protocol S1 Supplementary methods for determining functional traits.

Protocol S2 Comparison of the evaporative flux method and high-pressure method for determining K_{leaf}.

Protocol S3 Comparison of phylogenetic principal component analysis with linear discriminant analysis and canonical correlation analysis for data in this study.

Table S1. Phylogenetic clades, species names, and groups of the 42 species used in this study.

Table S2. Values for the 26 functional traits and six climatic niche descriptors of the 42 species used in this study. (In a separate Excel file)

Table S3. Raw data used to compare the evaporative flux method and high-pressure method for determining K_{leaf}. (In a separate Excel file)

Table S4. Pagel's λ for phylogenetic generalized least-squares models to analyse the effects of photosynthetic type and life history on plant traits, principal component scores and niche descriptors.

Fig. S1. Comparison of the evaporative flux method and high-pressure method to determine K_{leaf}.

Fig. S2. Images of leaf cross-sections of four typical species used in this study to determine K_{leaf}.

Fig. S3. Five functional traits for which photosynthetic type and life history models had similar explanatory power.

Protocol S1 Supplementary methods for determining functional traits.

Stomatal traits

Three images of epidermal peels were captured from each of three leaves per species. At 400× magnification we scored guard cell length (gl, μm) and width (gw, μm), calculating stomatal size (sts=gl×gw, μm^2). At 100× magnification we counted stomata in a 0.25 mm^2 field of view to determine stomatal density (std, mm^{-2}).

Seven of our 42 species were hypostomatous, and the abaxial and adaxial epidermes of the remaining 35 species were not significantly different in stomatal size (paired t_{110}=0.61, P=0.54) or density of stomata (paired t_{110}=1.73, P=0.09), hence our focus on stomatal traits of the abaxial surface in comparative analyses.

To integrate the effects of stomatal traits at the whole leaf level, we calculated maximum stomatal conductance to water vapour (g_{wmax}, mol m^{-2} s^{-1}) (Brown and Escombe, 1900; Franks and Beerling, 2009);

$$g_{\text{wmax}} = \frac{d}{v} \cdot \text{std} \cdot a_{\text{max}} \,/\, \left(l + \frac{\pi}{2}\sqrt{a_{\text{max}} \,/\, \pi}\right)$$

where d is the diffusivity of water vapour in air at 25 °C (m^2 s^{-1}); v is the molar volume of air at 25 °C (m^3 mol^{-1}); std is stomatal density; a_{max} is the maximum aperture of each stomatal pore, estimated as $\pi \cdot (p/2)^2$ where p is stomata pore length (Liu and Osborne, 2015). Pore length, p, is approximated as gl/2, and pore depth, l, as gw/2 (Franks and Beerling, 2009); π is the geometric constant. We calculated g_{wmax} for both leaf surfaces, and summed the values to obtain a value for the whole leaf.

Leaf and stem morphology

Leaf thickness (LT, μm) was determined as the average of tissue dimensions for major veins (thicker) and mesophyll (thinner), excluding the central vein. Leaf inter-vein distance (IVD, μm) was the distance between two adjacent leaf vein bundles from center to center, and diameter of leaf vein bundles (Dlvb, μm) was the average diameter of the major leaf vein bundles. Stem cross-sectional size (SS, mm^2) was calculated as an ellipse based on radii in long and short dimensions and subtracting any hollow areas from the centre of each stem (SS = $\pi \times r_{long} \times r_{short} - \pi \times r_{hollowlong} \times r_{hollowshort}$). Stem vessel density

(SVD, mm^{-2}) and the average diameter of stem vessels (Dsv, μm) were determined from two ~60-90° sectors (one in the long, and one in the short dimension of each stem). Stem vessel area proportion (VP) was the ratio of total vessel area [$\sum \pi \times (Dsv_i/2)^2$, $_i$ is the number of vessels] to each sector area (SS×$degree_{sector}$/360°), and was used to convert SS into stem vessel area in calculating K_S for each species [$K_S = K_h/(SS \times VP)$].

Leaf and stem hydraulics

Tillers were excised at their bases early in the morning. They were immediately re-cut underwater, placed into tubes of water in a bucket, covered with black plastic bags to prevent transpiration, and quickly transported to the laboratory.

For K_{leaf}, we found that ΔW_1, which is theoretically ideally zero, had an average value of $4.0\times10^{-8} \pm 4.0 \times10^{-8}$ kg s^{-1} over 60 s, i.e., 2.4 ± 2.4 mg.

Leaf pressure-volume curves

In the early morning, youngest mature leaves were placed under water and cut through the sheath, and were transferred to the lab in tubes full of water. The leaves were allowed to rehydrate in a bucket covered with black plastic bags for five to six hours (preliminary measurements showed that leaves were fully saturated after four to five hours). Rehydrated leaves were re-cut underwater, gently dried with tissue paper, and sealed into a pressure chamber to determine an initial water potential (Ψ_{ini}). Periodic measurements of Ψ and leaf fresh mass started if $\Psi_{ini} > -0.1$ MPa and continued till the leaf was wilted.

Phylogenetic tree

The phylogenetic tree for the 42 species in this study was extracted from a published super tree of Poaceae (*c.* 2600 species) (Edwards *et al.*, 2010). Thirty species exactly matched those used by Edwards *et al.* (2010) after considering synonymies (Clayton, 2002 onwards). Six other species were represented by a single congener in the super tree (*Brachiaria, Eriochloa, Ischaemum, Leptochloa, Sporobolus* and *Stenotaphrum*), and six species were settled as dichotomies between exactly matched congeners (one species in *Cyrtococcum, Eragrostis, Panicum* and *Paspalum*, two species in *Digitaria*). Polytomies and missing branch length information have only negligible impacts on phylogenetic

signals using phylogenetic generalized least squares (*PGLS*) (Stone, 2011; Münkemüller *et al.*, 2012). We transformed branch lengths of our phylogenetic tree using Grafen's method (Grafen, 1989) to avoid the effects of setting dichotomies.

Protocol S2 Comparison of the evaporative flux method and high-pressure method for determining K_{leaf}.

To validate the high-pressure method (HPM) for determining K_{leaf}, we made paired comparisons with K_{leaf} measured using evaporative flux method (EFM) (Sack and Scoffoni, 2012). These paired measurements used six common grasses representing C_3/C_4 and annual/perennial contrasts, sampled at the same location as the original study. Eight replicates were measured for each species, with one species being measured per day using both EFM and HPM.

In June 2018, 5~10 mature plants with roots were collected on the afternoon before measurements, into shallow water in a bucket covered with dark plastic bags. They were transported directly to the laboratory and allowed to rehydrate overnight. The next day, K_{leaf} was measured for one or two leaves per individual (10–20 leaves per species). Leaves with sheaths were recut under de-gassed distilled water using a fresh razor blade and, without overlapping the two edges, were wrapped around solid rods. Parafilm was wrapped around the rod and sheath, and then this sealed "petiole" was connected to a plastic tube linked to a cylinder filled with degassed pure water on an analytical balance (resolution ± 10 µg; Sartorius CPA225D; Sartorius; Göttingen, Germany). Leaves were positioned on a grid with their adaxial surface upward, and photosynthetically active radiation of 1200 µmol m^{-2} s^{-1} at the leaf surface was provided to stimulate transpiration. Air temperature in the air conditioned lab was around 25 °C. A transparent plastic tank filled with water was placed between the lamp and the leaf to absorb the heat from the lamp, and a fan was positioned near the leaf to increase the flow of air over its surface. Leaves were allowed to transpire for at least 20 min, and until the transpiration rate stabilized for at least 5 min, based on weights logged at a 30 s interval. Leaf temperature was recorded using a thermocouple once flux stabilized. Leaf transpiration rate (E; mmol m^{-2} s^{-1}) was calculated as the average of values during the stabilized period normalized by leaf area. The final leaf water potential (Ψ_{final}) was measured using a pressure chamber (Plant Moisture Systems, Corvallis, Oregon, USA) after 20 min equilibration in a sealed, humidified bag, in the dark. The pressure gradient ($\Delta\Psi_{leaf}$) driving water though the leaf was calculated as 0 MPa − Ψ_{final}. K_{leaf} (mmol m^{-2} s^{-1} MPa^{-1}) was calculated as $E/\Delta\Psi_{leaf}$,

standardized for the temperature dependence of viscosity of water to 25°C (Sack *et al.*, 2002).

Because in many species K_{leaf} declines with even a moderate leaf dehydration (Brodribb and Holbrook, 2003; Scoffoni *et al.*, 2012), we further standardized EFM measurements following Scoffoni *et al.* (2016). We regressed K_{leaf} against Ψ_{final} and calculated the maximum K_{leaf} as either: (1) the *y*-intercept of the regression line, if the slope was significant (five species); or (2) the average of all K_{leaf} values when the regression slope was not significant (one species).

EFM and HPM produced similar maximum K_{leaf} values, with a slope that was not significantly different from 1:1 (Fig. S1). These results are consistent with comparisons among three K_{leaf} measurement methods applied to leaves of eudicot woody species (Sack *et al.*, 2002).

Pressurization in the HPM may force water to move not only through the xylem, but also through outside-xylem pathways, including aerenchyma, which could provide very low resistance for axial water transport. For our 42 studied grass species, none were selected from aquatic or very wet habitats where species would be most likely to form aerenchyma, which usually occurs in roots, stems, in some cases in leaf sheaths or petioles, but not in leaves (Jackson and Armstrong, 1999; Evans, 2004). Below, we provided leaf cross-section images of four typical C_3/C_4 and annual/perennial grass species, which did not show aerenchyma tissues (Fig. S2). We also never observed large amounts of water flow during HPM measurements; by contrast with eudicot leaves, to obtain sufficient mass for measurement it was necessary to collect flow solution from grass leaves in 60 s instead of the more usual 10-30 s. We therefore expected that impacts of aerenchyma tissues on HPM results are unlikely, at least for the 42 grass species used in this study.

Protocol S3 Comparison of phylogenetic principal component analysis with linear discriminant analysis and canonical correlation analysis for data in this study.

Linear Discriminant Analysis (LDA) and Phylogenetic Canonical Correlation Analysis (PCCA) provide alternative, albeit differently motivated, multivariate decompositions of our dataset, compared with the Phylogenetic Principal Component Analysis (PPCA) reported in the main text. We found that LDA showed similar results to PPCA. PCCA supported several functional linkages between structural and physiological traits, reported here as Supplementary Data because our manuscript focused on associations with life history and photosynthetic type rather than trait-trait linkages (Fig. R1-R4, Table R1-R2).

Linear Discriminant Analysis (LDA), which sets prior groupings and creates new variables (LDs) by maximizing difference between groups, was implemented using the function *lda* in R package *MASS*. As yet, no method for LDA is provided that accounts for phylogenetic covariance, but we found results that were similar to those for PPCA, for both the 11 physiological traits (Fig. R1) and the 15 structural traits (Fig. R2).

Phylogenetic Canonical Correlation Analysis (PCCA) was implemented using the function *phyl.cca* in R package *phytools*. PCCA identifies important axes that describe among-replicate variation common among separate multivariate datasets of the same samples; in our analysis it is relevant to understanding covariation between structural and physiological traits. We found 11 canonical dimensions, of which the first three were statistically significant at $P<0.05$. Dimension 1 had a canonical correlation of 0.97 between the two sets of variables, while for dimension 2 and 3 the canonical correlation was at 0.95 and 0.94 respectively (Table R1). The first canonical dimension linked stem vessel density (SVD, 0.89), leaf to stem area ratio (AlAs, -0.68) and diameter of stem vessels (Dcv, 0.59) (structural traits) with variation in $K_{svessel}$ (-1.49), K_L (1.48), A (-1.0) and g_s (0.86) (physiological traits). The second dimension linked stomatal density (sd, -1.30) and stomatal size (ssize, -0.91) (structural traits) with WUE_i (3.98), g_s (3.33) and A (-3.29) (physiological traits) (Table R2; Fig. R3-R4).

These results are interesting, because they suggest that covariation among the structural traits we measured explained only some of the covariation in the physiological traits captured by our PCA analyses. Among structural traits, the SVD, AlAs, Dcv axis is

consistent with the first PCA, but WUE_i, g_s, A, $K_{svessel}$ and K_L separated from water potential traits in PCA of physiological traits. This highlights an important point that the set of traits included in a study determine the insights from PCCA. Because our goal was to establish whether functional categories corresponded with differentiation in traits, we chose to measure traits that we anticipated would have broad relevance to hydraulic performance and fast-slow strategies. We did not choose traits on the basis of expectations about their functional linkages. For example, the set of structural traits we chose do not have strong associations with K_{leaf}, but variation in this physiological trait is important when we look at the how PPCA scores separated among functional types. Furthermore, because C_4 physiology in grasses requires both anatomical and biochemical modifications, we would not anticipate that structure-function relationships would be absolutely parallel across C_3 and C_4 plants. An ideal analysis might consider trait linkages within C_3 and C_4 species separately.

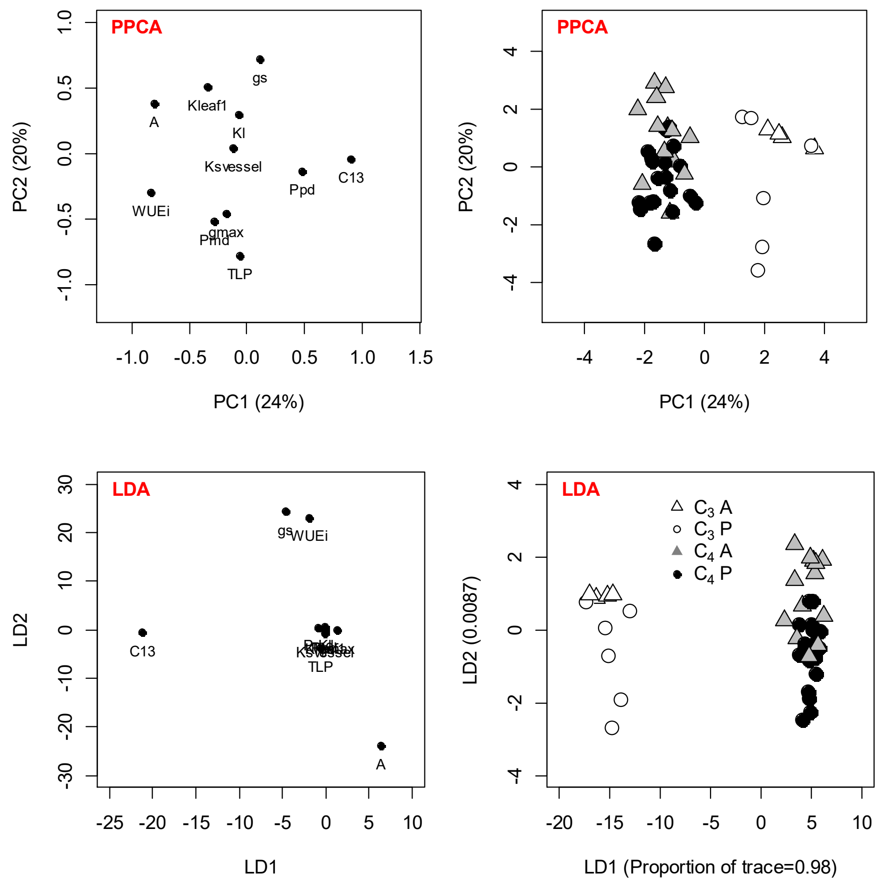

Fig. R1. Comparions of PPCA and LDA for 11 physiological traits. Left column, trait loadings of PPCA or scalings of LDA; right column, species scores. Trait loadings showed similar pattern between LDA and PPCA, with A, δ^{13}C, WUE_i and g_s the four traits that drove the species grouping. C_3 and C_4 species were clearly distinguished along the first axis (x-axis) in both analyses, though annuals and perennials were a little better discriminated along the second axis (y-axis) for LDA than for PPCA.

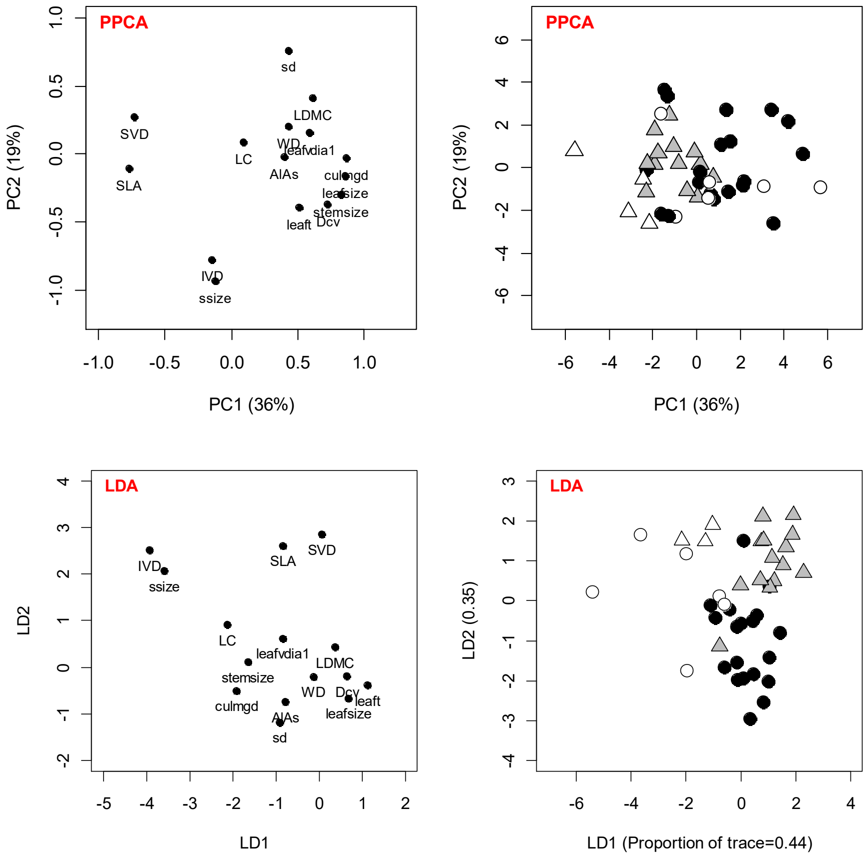

Fig. R2. Comparions of PPCA and LDA for 15 structural traits. Left column, trait loadings of PPCA or scalings of LDA; right column, species scores. PPCA and LDA again had similar results. SVD (stem vessel density) and SLA were the main drivers to discriminate annuals and perennials, while IVD (leaf interveinal distance) and ssize (stomatal size) modestly distinguish C_3 and C_4 species. However, LDA did not provide a much greater separation of species along the x and y axes compared with PPCA.

Table R1. Tests of Canonical Dimensions

Dimension	Corr.	*F*	df1	df2	*p*
**1**	0.97	2.47	165	164.2	**0.0000**
**2**	0.95	1.96	140	156.7	**0.0000**
**3**	0.94	1.53	117	148.0	**0.0070**
4	0.76	1.10	96	138.2	0.3065
5	0.74	1.05	77	127.3	0.4047
6	0.70	0.97	60	115.1	0.5410
7	0.68	0.88	45	101.5	0.6718
8	0.56	0.71	32	86.4	0.8575
9	0.53	0.62	21	69.5	0.8804
10	0.31	0.36	12	50.0	0.9711
11	0.25	0.35	5	26.0	0.8799

Table R2. Standardized Canonical Coefficients

	Dimension 1	Dimension 2	Dimension 3
Structural traits			
culmgd	0.07	-0.06	0.26
ssize	0.41	-0.91	-0.49
sd	0.50	-1.30	-0.02
leafsize	-0.16	-0.19	-0.45
leaft	0.01	0.02	-0.03
IVD	0.09	-0.30	0.93
leafvdia1	0.03	0.09	-0.23
stemsize	0.09	-0.16	0.58
Dcv	0.59	0.20	-0.12
SLA	0.12	-0.06	0.29
WD	-0.04	-0.15	-0.16
LDMC	0.12	-0.28	0.65
AlAs	-0.68	-0.01	0.40
LC	-0.07	0.22	-0.21
SVD	0.89	-0.29	0.67
Physiological traits			
A	-1.00	-3.29	-0.96
gs	0.86	3.33	1.07
WUEi	0.84	3.98	1.35
gmax	0.16	-0.74	-0.58
Kleaf1	-0.08	0.07	-0.16
Ksvessel	-1.49	-0.08	-0.06
TLP	0.07	-0.27	0.41
Ppd	0.15	-0.13	0.32
Pmd	0.11	-0.04	0.14
Kl	1.48	0.15	-0.27
C13	-0.13	0.01	0.65

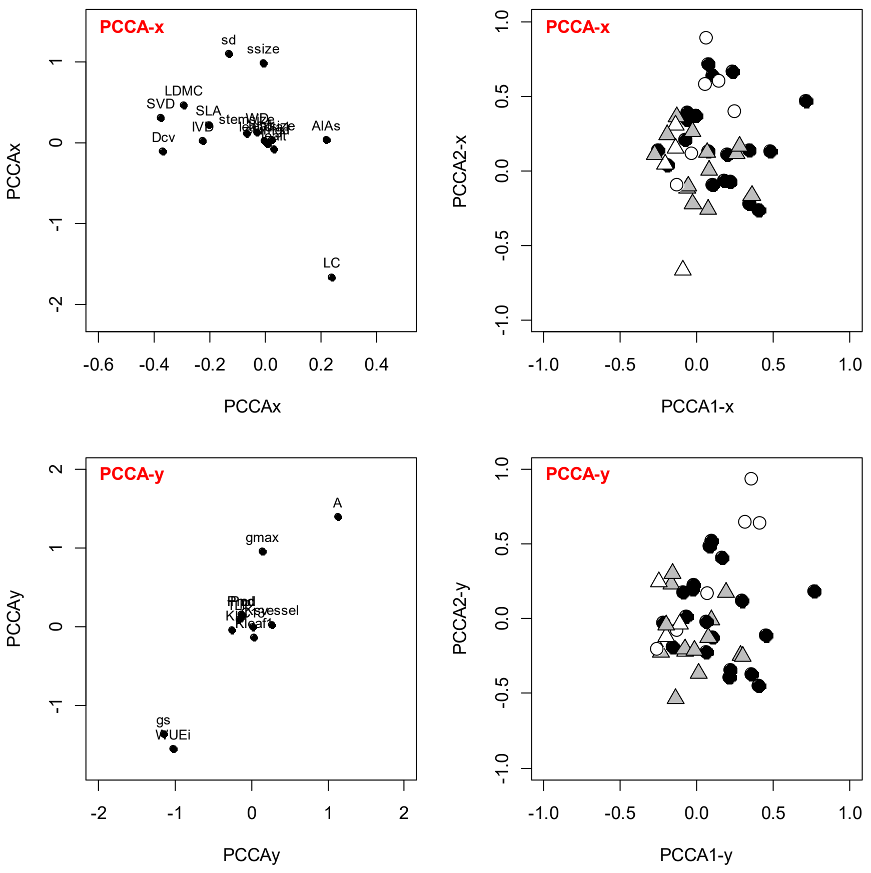

Fig. R3. PCCA between 15 structural traits (X) and 11 physiological traits (Y). Left column, trait estimated coefficients for the canonical variables; right column, species canonical scores. Symbols are as Fig. R1.

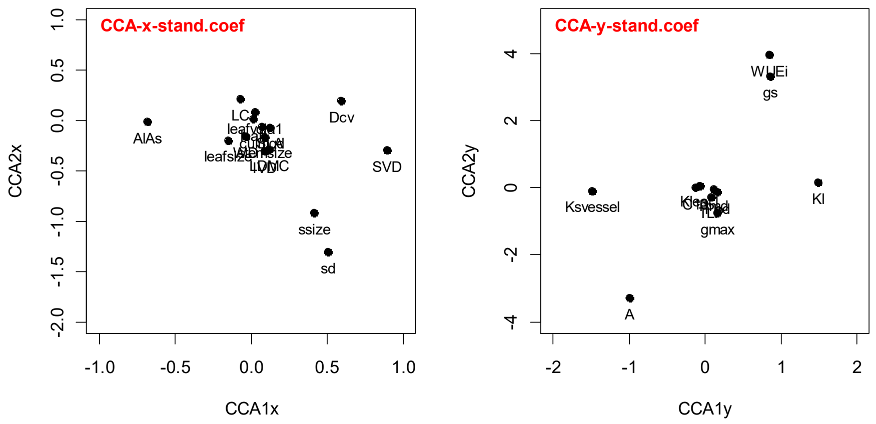

Fig. R4. Standardized Canonical Coefficients of dimensions 1 and 2 of CCA between 15 structural traits (X) and 11 physiological traits (Y).

Table S1. Phylogenetic clades, species names and groups of the 42 species used in this study. PT, photosynthetic type; Life history: A, annuals, and P, perennials.

Clade	Species	PT	AP
Arundinoideae	*Phragmites australis* Trin. ex Steud.	C_3	P
Chloridoideae, Cynodonteae	*Cynodon dactylon* (Linn.) Pers.	C_4	P
	Dactyloctenium aegyptium (Linn.) Beauv.	C_4	A
	Eleusine indica (Linn.) Gaertn.	C_4	A
	Leptochloa chinensis (Linn.) Nees	C_4	A
Chloridoideae, Eragrostideae	*Eragrostis atrovirens* (Desf.) Trin. ex Steud.	C_4	P
	Eragrostis perlaxa Keng	C_4	P
Chloridoideae, Triraphidieae	*Neyraudia reynaudiana* (Kunth) Keng ex Hithc.	C_4	P
Chloridoideae, Zoysieae	*Sporobolus hancei* Rendle.	C_4	P
Panicoideae, Andropogoneae	*Apluda mutica* Linn.	C_4	P
	Bothriochloa ischaemum (Linn.) Keng	C_4	P
	Imperata cylindrica (Retz.) Beauv.	C_4	P
	Ischaemum barbatum Retz.	C_4	P
	Microstegium vimineum (Trin.) A. Camus	C_4	A
	Miscanthus floridulus (Lab.) Warb. ex K. Schum.et Laut.	C_4	P
Panicoideae, Centotheceae	*Centotheca lappacea* (Linn.) Desv.	C_3	P
Panicoideae, Paniceae	*Brachiaria subquadripara* (Trin.) Hitchc.	C_4	A
	Cyrtococcum accrescens (Trin.) Stapf	C_3	A
	Cyrtococcum patens (Linn.) A. Camus.	C_3	A
	Digitaria chrysoblephara Fig.	C_4	A
	Digitaria henryi Rendle	C_4	A
	Digitaria violascens Link.	C_4	A
	Echinochloa colonum (Linn.) Link.	C_4	A
	Echinochloa crusgalli var. *breviseta* (Linn.) P. Beauv. (Doell.) Neilr.	C_4	A
	Eriochloa procera (Retz.) Hubb.	C_4	A
	Megathrysus maximus	C_4	P
	Oplismenus compositus (Linn.) Beauv.	C_3	P
	Ottochloa nodosa (Kunth) Dandy	C_3	P

	Panicum repens Linn.	C_4	P
	Pennisetum purpureum Schum.	C_4	P
	Sacciolepis indica (Linn.) A. Chase	C_3	A
	Setaria glauca (Linn.) Beauv.	C_4	A
	Setaria palmifolia (Koen.) Stapf	C_4	P
	Setaria viridis (Linn.) Beauv.	C_4	A
	Stenotaphrum helferii Munro ex Hook. f.	C_4	P
Panicoideae, Paspaleae	*Axonopus compressus* (Sw.) Beauv.	C_4	P
	Paspalum conjugatum Berg.	C_4	P
	Paspalum dilatatum Poir.	C_4	P
	Paspalum orbiculare Forst.	C_4	P
Panicoideae, Thysanolaeneae	*Thysanolaena latifolia* (Roxb.) Ktze.	C_3	P
Panicoideae, Zeugiteae	*Lophatherum gracile* Brongn	C_3	P
Pooideae, Poeae	*Alopecurus aequalis* Sobol.	C_3	A

Table S4. Pagel's λ for phylogenetic generalized least-squares models to analyse the effects of photosynthetic type (PT, C_3 and C_4) and life history (AP, annual and perennial) on (a) plant functional traits (42 species), (b) principal component scores for trait variation, (c) niche descriptors (34 species, due to the lack of climatic data of eight species), and (d) principal component scores for niche descriptor variation. Models used $\log_e$-transformed values (absolute values were input for measurements normally expressed as negative values) except for principal component scores.

	Pagel's λ			
	AP×PT	AP+PT	PT	AP
(a) Plant functional traits				
IVD (μm)	0.00	0.00	0.00	0.46
SD (g cm^{-3})	0.00	0.00	0.43	0.00
$-\Psi_{tlp}$ (MPa)	0.00	0.00	0.26	0.00
Leaf δ^{13}C (‰)	0.62	0.62	0.62	1.00
K_{leaf} (mmol m^{-2} s^{-1} MPa^{-1})	0.24	0.22	0.23	0.17
$-\Psi_{pre}$ (MPa)	0.00	0.00	0.00	0.00
SLA (cm^2 g^{-1})	0.48	0.50	0.51	0.52
Dsv (μm)	0.00	0.00	0.10	0.00
g_s (mol m^{-2} s^{-1})	0.32	0.32	0.56	0.36
Dlvb (μm)	0.00	0.00	0.00	0.00
sts (μm^2)	0.26	0.20	0.20	0.27
H (cm)	0.00	0.00	0.33	0.00
LDMC (%)	0.04	0.06	0.16	0.08
A_L/A_S (cm^2 mm^{-2})	0.00	0.00	0.00	0.00
LT (μm)	0.00	0.00	0.00	0.00
g_{wmax} (mol m^{-2} s^{-1})	0.43	0.58	0.61	0.57
LA (cm^2)	0.09	0.24	0.50	0.17
SS (mm^2)	0.00	0.00	0.00	0.00
WUE$_i$ (μmol mol^{-1})	0.25	0.25	0.45	0.94
LC (%)	0.00	0.00	0.00	0.00
std (mm^{-2})	0.03	0.05	0.08	0.09

K_S (kg m^{-1} s^{-1} MPa^{-1})	0.00	0.00	0.00	0.00
A (μmol m^{-2} s^{-1})	0.00	0.00	0.00	0.94
$-\Psi_{mid}$ (MPa)	0.00	0.00	0.00	0.00
SVD (mm^{-2})	0.00	0.00	0.00	0.00
K_L (10^{-4} kg m^{-1} s^{-1} MPa^{-1})	0.51	0.50	0.50	0.51
(b) Principal component scores for trait variation				
PC2 of physiological traits	0.50	0.44	0.56	0.44
PC1 of physiological traits	0.00	0.00	0.00	0.98
PC1 of structural traits	0.00	0.00	0.38	0.00
PC1 of all traits	0.00	0.00	0.56	0.00
(c) Niche descriptors				
Precipitation seasonality	0.46	0.46	0.28	0.50
Temperature seasonality	0.00	0.00	0.00	0.00
MAP (mm)	0.00	0.00	0.31	0.00
Wet days per year	0.00	0.00	0.20	0.00
MAT (°C)	0.88	0.88	0.92	0.88
Tree cover (%)	0.00	0.00	0.10	0.00
(d) Principal component scores for niche variation				
PC1 of six niche descriptors	0.00	0.00	0.00	0.00
PC2 of six niche descriptors	0.59	0.59	0.53	0.66

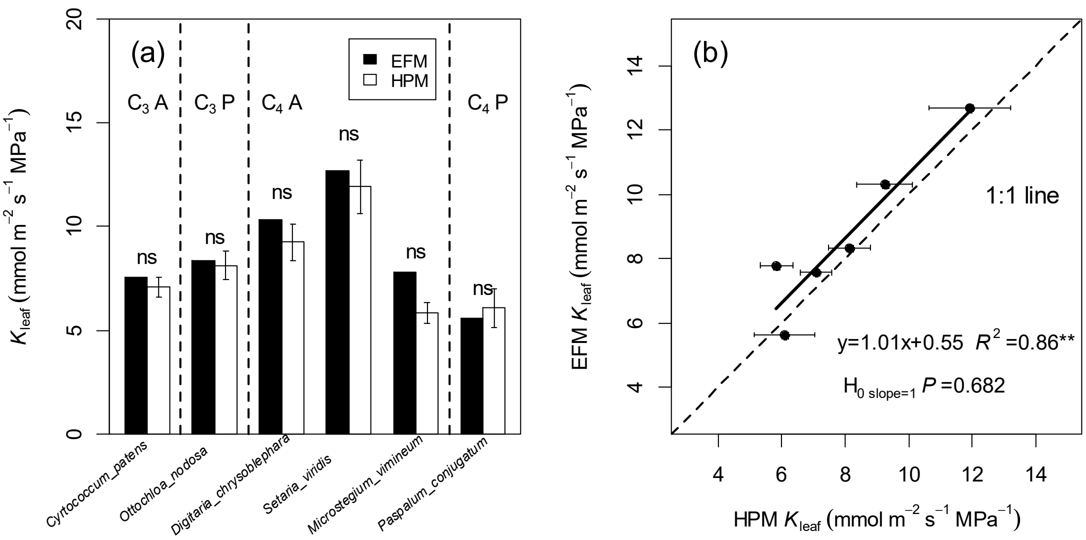

Fig. S1. Comparisons of the evaporative flux method (EFM) and high-pressure method (HPM) to determine K_{leaf}. (a) Paired bars comparing EFM and HPM measurements of K_{leaf} (ns, non-significant) for six species representative of key groups in the original experiments. (b) Comparison of K_{leaf} EFM-HPM regression (solid line) with 1:1 line (dashed line). For HPM data, error bars in (a) and (b) are standard errors of 8 replicates, while for EFM data, maximum K_{leaf} is calculated based on 10-11 leaf measurements.

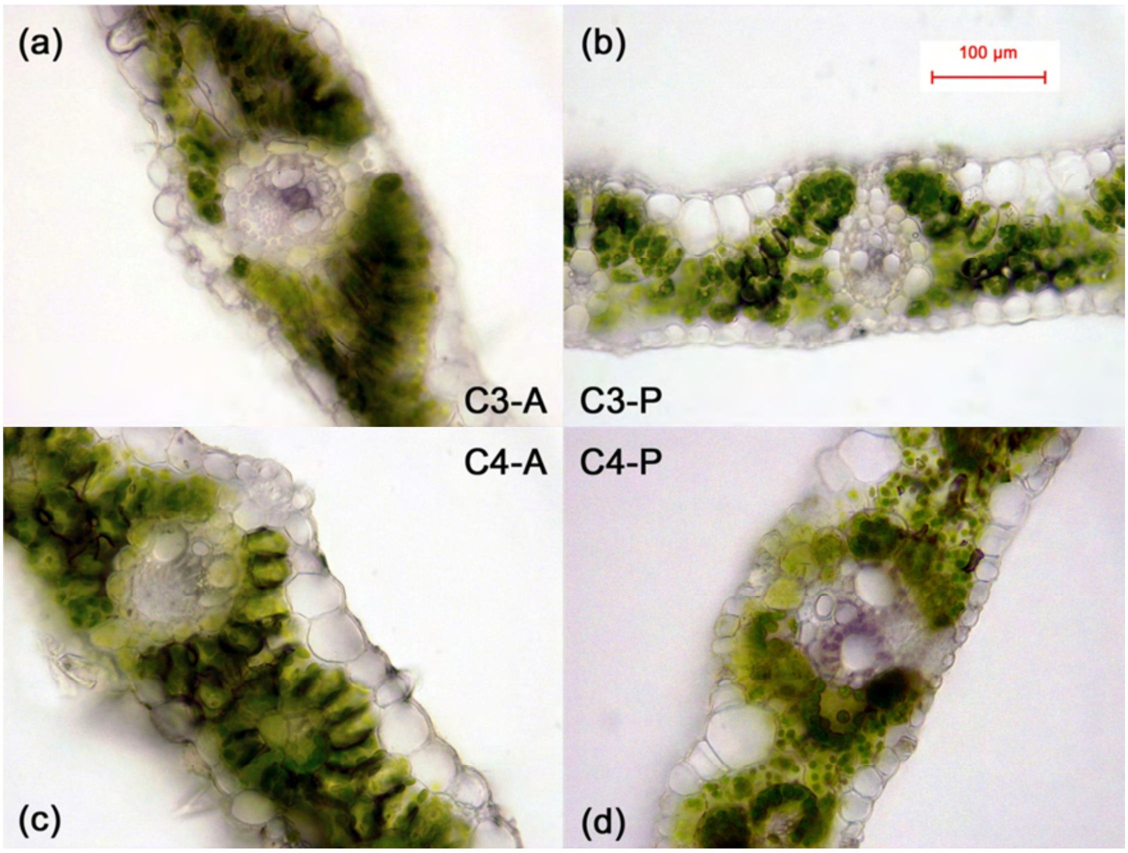

Fig. S2. Images of leaf cross-sections of four typical species used in this study to determine K_{leaf}. (a) *Cyrtococcum patens* (C_3-A); (b) *Ottochloa nodosa* (C_3-P); (c) *Digitaria chrysoblephara* (C_4-A); and (d) *Imperata cylindrica* (C_4-P). Notice that species (d) was not used in Fig. S1 for the comparisons between two K_{leaf} measurement methods.

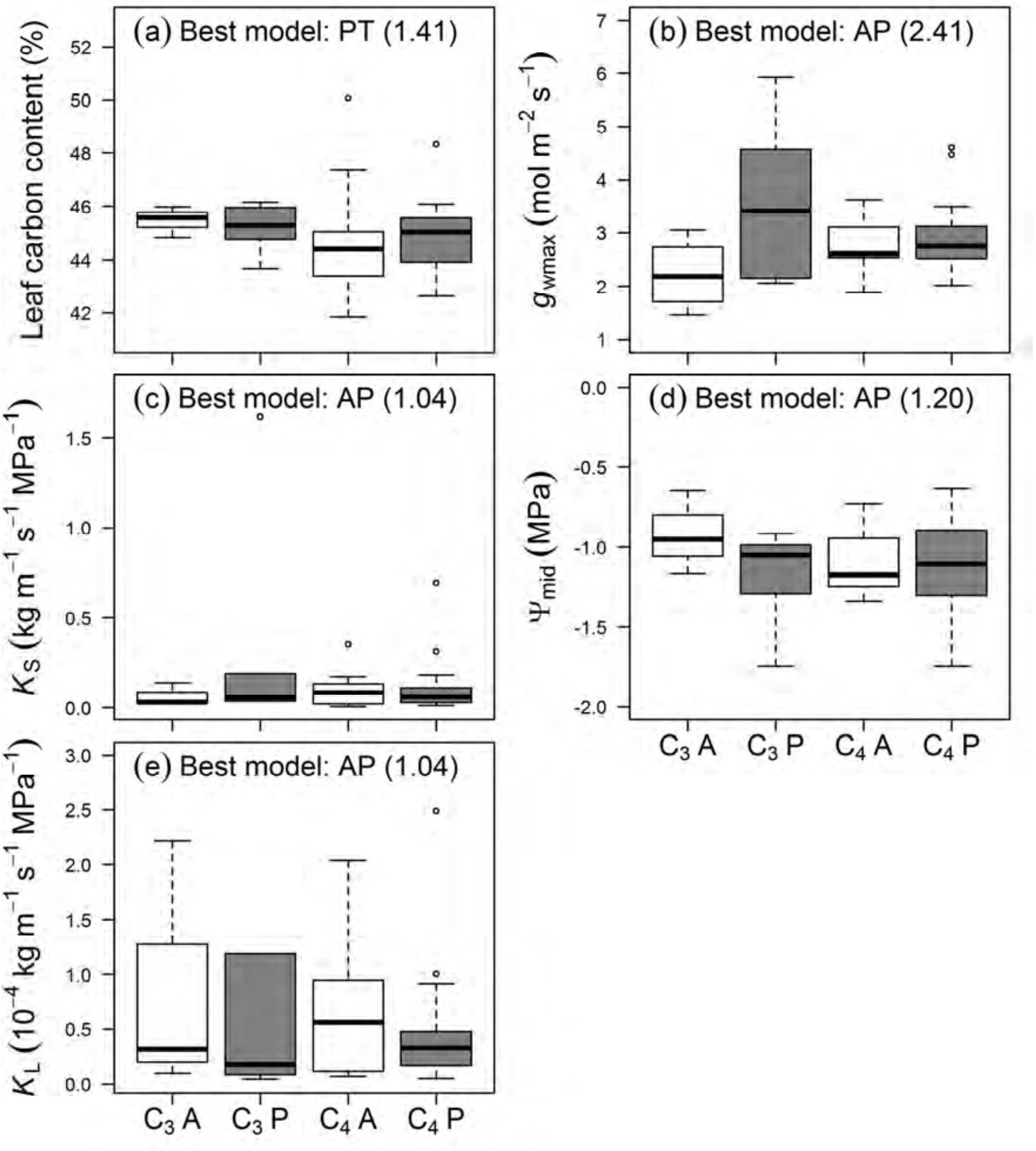

Fig. S3. Five functional traits for which AP (annual, open boxes; perennial, grey boxes) and PT (C_3, left; C_4, right) models had similar explanatory power. The box plots show quartiles for each trait with extreme values as circles. Sample size for C_3-A, C_3-P, C_4-A and C_4-P are 4, 6, 13 and 19, respectively. The best-fit model with evidence ratio is reported for each trait.

References

Brodribb TJ, Holbrook NM. 2003. Stomatal closure during leaf dehydration, correlation with other leaf physiological traits. *Plant Physiology* **132**, 2166-2173.

Brown HT, Escombe F. 1900. Static diffusion of gases and liquids in relation to the assimilation of carbon and translocation in plants. *Proceedings of the Royal Society of London* **67**, 124-128.

Clayton WD, Vorontsova, M.S., Harman, K.T. and Williamson, H. 2002 onwards. World Grass Species: Synonymy. http://www.kew.org/data/grasses-syn.html. [accessed September 2014].

Edwards EJ, Osborne CP, Stromberg CAE, Smith SA, Consortium CG. 2010. The origins of C_4 grasslands: Integrating evolutionary and ecosystem science. *Science* **328**, 587-591.

Evans DE. 2004. Aerenchyma formation. *New Phytologist* **161**, 35-49.

Franks PJ, Beerling DJ. 2009. Maximum leaf conductance driven by CO_2 effects on stomatal size and density over geologic time. *Proceedings of the National Academy of Sciences* **106**, 10343-10347.

Grafen A. 1989. The phylogenetic regression. *Philosophical Transactions of the Royal Society of London B Biological Sciences* **326**, 119-157.

Jackson M, Armstrong W. 1999. Formation of aerenchyma and the processes of plant ventilation in relation to soil flooding and submergence. *Plant Biology* **1**, 274-287.

Liu H, Osborne CP. 2015. Water relations traits of C_4 grasses depend on phylogenetic lineage, photosynthetic pathway, and habitat water availability. *Journal of Experimental Botany* **66**, 761-773.

Münkemüller T, Lavergne S, Bzeznik B, Dray S, Jombart T, Schiffers K, Thuiller W. 2012. How to measure and test phylogenetic signal. *Methods in Ecology and Evolution* **3**, 743-756.

Sack L, Melcher PJ, Zwieniecki MA, Holbrook NM. 2002. The hydraulic conductance of the angiosperm leaf lamina: a comparison of three measurement methods. *Journal of Experimental Botany* **53**, 2177-2184.

Sack L, Scoffoni C. 2012. Measurement of Leaf Hydraulic Conductance and Stomatal Conductance and Their Responses to Irradiance and Dehydration Using the Evaporative Flux Method (EFM). *Journal of Visualized Experiments*, 4179.

Scoffoni C, Chatelet DS, Pasquet-kok J, Rawls M, Donoghue MJ, Edwards EJ, Sack L. 2016. Hydraulic basis for the evolution of photosynthetic productivity. *Nature Plants* **2**.

Scoffoni C, McKown AD, Rawls M, Sack L. 2012. Dynamics of leaf hydraulic conductance with water status: quantification and analysis of species differences under steady state. *Journal of Experimental Botany* **63**, 643-658.

Stone EA. 2011. Why the phylogenetic regression appears robust to tree misspecification. *Systematic Biology* **60**, 245-260.
